# Biomedical Applications of *Scutellaria edelbergii* Rech. f.: In Vitro and In Vivo Approach

**DOI:** 10.3390/molecules26123740

**Published:** 2021-06-19

**Authors:** Muddaser Shah, Waheed Murad, Najeeb Ur Rehman, Sobia Ahsan Halim, Manzoor Ahmed, Hazir Rehman, Muhammed Zahoor, Sidra Mubin, Ajmal Khan, Mohamed A. Nassan, Gaber El-Saber Batiha, Ahmed Al-Harrasi

**Affiliations:** 1 Department of Botany, Abdul Wali Khan University Mardan, Mardan 23200, Pakistan; muddasershah@awkum.edu.pk; 2 Natural and Medical Sciences Research Center, University of Nizwa, P.O. Box 33, Birkat Al Mauz, Nizwa 616, Oman; sobia_halim@unizwa.edu.om (S.A.H.); ajmalkhan@unizwa.edu.om (A.K.); 3 Department of Chemistry, University of Malakand, Chakdara 18800, Pakistan; manzoorhej@yahoo.com; 4 Department of Microbiology, Abdul Wali Khan University Mardan, Mardan 23200, Pakistan; hazirrahman@awkum.edu.pk; 5 Department of Biochemistry, University of Malakand, Chakdara 18800, Pakistan; mohammadzahoorus@yahoo.com; 6 Department of Botany, Hazara University Mansehra, Mansehra 21310, Pakistan; shahhu123@gmail.com; 7 Department of Clinical Laboratory Sciences, Turabah University College, Taif University, P.O. Box 11099, Taif 21944, Saudi Arabia; m.nassan@tu.edu.sa; 8 Department of Pharmacology and Therapeutics, Faculty of Veterinary Medicine, Damanhour University, Damanhour 22511, Egypt; gaberbatiha@gmail.com

**Keywords:** *Scutellaria edelbergii* Rech. f., natural products, antimicrobial, antioxidant, anti-inflammatory, analgesic assays

## Abstract

In the current study, in vitro antimicrobial and antioxidant activities and in vivo anti-inflammatory and analgesic activities of *Scutellaria edelbergii* Rech. f. (crude extract and subfractions, i.e., n-hexane, ethyl acetate (EtOAc), chloroform, n-butanol (n-BuOH) and aqueous) were explored. Initially, extraction and fractionation of the selected medicinal plant were carried out, followed by phytochemical qualitative tests, which were mostly positive for all the extracts. EtOAc fraction possessed a significant amount of phenolic (79.2 ± 0.30 mg GAE/g) and flavonoid (84.0 ± 0.39 mg QE/g) content. The EtOAc fraction of *S. edelbergii* exhibited appreciable antibacterial activity against Gram-negative (*Escherichia coli* and *Klebsiella pneumoniae*) strains and significant zones of inhibition were observed against Gram-positive bacterial strains (*Bacillus subtilis* and *Staphylococcus aureus*). However, it was found inactive against *Candida Albicans* and *Fusarium oxysporum* fungal strains. The chloroform fraction was the most effective with an IC_50_ value of 172 and 74 µg/mL against DPPH (1,1-Diphenyl-2-picryl-hydrazyl) and ABTS assays, in comparison with standard ascorbic acid 59 and 63 µg/mL, respectively. Moreover, the EtOAc fraction displayed significant in vivo anti-inflammatory activity (54%) using carrageenan-induced assay and significant (55%) in vivo analgesic activity using acetic acid-induced writing assay. In addition, nine known compounds, ursolic acid (**UA**), ovaul (**OV**), oleanolic acid (**OA**), β-sitosterol (**BS**), micromeric acid (**MA**), taraxasterol acetate (**TA**), 5,3′,4′-trihydroxy-7-methoxy flavone (**FL-1**), 5,7,4′-trihydroxy-6,3′-dimiethoxyflavone (**FL-2**) and 7-methoxy catechin (**FL-3**), were isolated from methanolic extract of *S. edelbergii*. These constituents have never been obtained from this source. The structures of all the isolated constituents were elucidated by spectroscopic means. In conclusion, the EtOAc fraction and all other fractions of *S. edelbergii*, in general, displayed a significant role as antibacterial, free radical scavenger, anti-inflammatory and analgesic agents which may be due to the presence of these constituents and other flavonoids.

## 1. Introduction

More than 10% of the 32,000 reported species of higher plants are of medicinal importance worldwide [1,2,3]. In developing countries, synthetic pharmaceutical drugs are expensive and not affordable for an average person; therefore, the plants are the primary source of phytomedicines for inflammation, pain and microbial resistance [4]. Herbal medicines are important to enhance clinical research and quality control along with treatment and prevention of disease [5]. The demands for herbal medicines are frequently increasing and can be met by screening them in vitro biological and in vivo pharmacological features. The investigation of ethnomedicinal plants is required in developing nations due to costly allopathic medicines [5,6]. Therefore, it is essential to determine the significance of plants and enhance plant medication knowledge using modern approaches as they are less toxic and more effective for humanity [7].

One of the important human health issues is the drug resistance developed by microbes against the available antimicrobial drugs and it has been reported that within next 30 years, existing drugs will be totally inactive against them [8,9,10]. Consequently, there is a need to investigate plants for new therapeutic agents as plant-derived drugs are usually associated with fewer side effects in comparison to drugs prepared through synthetic routes [9].

Oxidative stress is another health issue prevalent these days. Several oxygen-based free radicals are usually formed during normal physiological and biochemical processes taking place in human bodies that are immediately detoxified by the human defense system immediately. However, in the past few decades, human food habits have changed drastically [10] and dependence on synthetic ingredients in foods has increased, which has given rise to the overproduction of free radicals in human bodies. Several chronic disorders, such as atherosclerosis, inflammations, cancer, diabetes, pain, aging and other degenerative complications in humans, are due to oxidative stress [4]. Plants and plant products (fruits, vegetables, medicinal herbs) contain various phenolic compounds that can scavenge free radicals as they have aromatic rings which can stabilize the free radicals formed inside body and in vitro [11]. The intake of natural antioxidants reduces the chances of cancer, cardiovascular abnormalities, dermatological infections, diabetes and other acute or chronic infections linked with aging [12,13].

The research on antioxidants from plant origins has also revealed that several antioxidants are effective in treating health complication associated with oxidative stress. Biological potentials such as anti-inflammatory, analgesic, anti-cancer, antipyretic and antibacterial activities of some antioxidants from plant origin are well documented [12]. Regardless of the latest developments in pain therapies, scientists still want to explore safe, effective and potent analgesic medication from plant origin as they are associated with fewer side effects, as mentioned earlier [14,15].

*Scutellaria* is a genus of the family Lamiaceae, also termed as skullcap and mostly found in temperate regions [16]. Generally, the genus is a rich source of scutellarin, baicalin, alkaloids, saponins, tannins and glycosides, having significant in vivo pharmacological effects in anti-inflammatory, analgesic, antibacterial, antifungal and antioxidant potentials [17,18,19]. *Scutellaria edelbergii* dried leaves powder is used as a blood purifier and an antimicrobial, anti-inflammatory and analgesic agent. Traditionally, its leaves are also used as green tea (kava) as it has medicinal importance. The extracts of *S. baicalensis* and its major chemical constituents have been reported to possess anti-viral, anti-tumor, anti-bacterial, antioxidant, anti-inflammatory, hepatoprotective and neuroprotective activities [17,20]. In addition, 126 molecules including flavonoids, flavones, isoflavones and six polysaccharides have been isolated from *S. baicalensis* [20]. The dried herb of *S. indica* is a crude drug (known as han xin cao in China) that has been used as anti-analgesic, antidotic and hemostatic for the treatment of hemoptysis, hematemesis and other diseases [18]. *S. edelbergii* have not been studied before for the mentioned pharmacological properties.

To attract the attention of researchers, the ongoing investigations aimed to provide information about the effectiveness of selected plants in the number of health complications and revive the significance of natural products in several health complications in general. *S. edelbergii* was subjected to extraction and fractionation followed by phytochemical investigation using different techniques. The extracts were also evaluated for antibacterial, antifungal and antioxidant potential in vitro; also, in vivo pharmacological activities such as analgesic and anti-inflammatory were also carried out.

## 2. Results and Discussion

Based on information collected from local inhabitants of Kalam, District Swat, Pakistan, the selected plant crude extract and subfractions (n-hexane, EtOAc, chloroform, n-BuOH, aqueous) were investigated for their phytochemical, in vitro antibacterial, antifungal and antioxidant potentials while in vivo pharmacological (anti-inflammatory and analgesic) activities were determined in Swiss albino mice using standard protocols. As *S. edelbergii* has not been investigated for the mentioned phytochemical, in vitro biological and in vivo pharmacological potentials, this study was conducted to promote the folkloric uses of the selected plant and provide scientific validation of *S. edelbergii* in terms of its phytochemical composition and observed biological potentials. In modern methods of isolating drugs from plants, ethnopharmacological information was collected from local practitioners and communities, then phytochemical analysis was performed and finally pharmacological studies were carried out to confirm the claimed medicinal potentials. The in vitro and in vivo investigation are basic approaches that expose the therapeutic significance of a given plant [19,21,22,23,24].

### 2.1. Qualitative Phytochemical Analysis of Extracts

Phytochemical analysis was performed to determine the chemical groups in the crude extract and their sub-fractions. All the extracts have shown the presence of flavonoids, phenols, alkaloids, saponins, carbohydrates and glycosides. The qualitative phytochemical tests were carried out to confirm the representative groups of phytochemicals in the tested extracts. Most of the fractions were positive for the applied tests (Table 1). Our results are in complete agreement with the reported data of *S. havanensis* [25].

### 2.2. Phenolic and Flavonoids Contents

The quantitative estimation of phenolic and flavonoid contents in crude extract and subfractions are presented in Table 2. The highest numbers of phenolic compounds were present in EtOAc fraction, crude extract and n-BuOH fractions. Comparable amounts of phenolic compounds were also found in the chloroform fraction. As phenolic compounds are polar, its high amount was therefore present in polar solvents. Nearly the same trend was observed for flavonoid contents; however, their amount was high as compared to phenolic contents. Similarly, the highest number of flavonoids was present in the EtOAc fraction, crude extract, n-BuOH and chloroform fractions. Both the phenolic and flavonoid contents were compared with standards and statistically analyzed at *p* ≤ 0.01 (Table 2). The EtOAc fraction was the richest fraction amongst the tested extracts. About 79.2 ± 0.30 mg GAE/g of total phenolic contents and 84.0 ± 0.39 mg QE/g total flavonoid contents were estimated in it. Our findings are in complete agreement with the study of Mousavi et al. [6], where *Scutellaria platystegia* was investigated for the mentioned contents. Higher phenolic and flavonoid contents were reported in *S. platystegia, S. barbata* and *S. orientalis,* belonging to the same genus [26,27].

### 2.3. Antibacterial Activity

The antibacterial potential of different extracts was tested against two Gram-negative (*E. coli* and *K. pneumonia*) and two Gram-positive (*S. aureus* and *B. subtilis*) bacterial strains along with standards and negative control (Table 3). Among the tested extracts, the EtOAc (SEEA) fraction exhibited the highest inhibitory potential for Gram-negative bacteria. At 50 µL and 100 µL, SEEA produced a zone of inhibition (ZOI) of 14.5 ± 0.04 and 17.9 ± 0.03 mm against *E. coli* and 14.3 ± 0.02 and 17.3± 0.02 mm against the *K. pneumonia*, respectively. Comparatively, *K. pneumoniae* was highly susceptible to *S. edelbergii* extracts. Moreover, SEEA was also found most effective against *S. aureus* and *B. subtilis* with the zone of inhibition of 15.03 ± 0.01 and 19.07 ± 0.02 mm and 4.97 ± 0.01 and 18.87 ± 0.04 mm at 50 µL and 100 µL, respectively. Moreover, all the fractions were found active in the antibacterial assay. The results are summarized in Table 3.

Appreciable inhibition potentials were observed for all fractions against tested bacterial strains. The EtOAc fraction was found to be the most active fraction with 19.07 ± 0.02 and 17.09 ± 0.01 mm zones of inhibitions against *S. aureus* and *B. subtilis*, respectively, at high doses only. The chloroform fraction exhibited zones of inhibition of 17.03 ± 0.03 and 17.09 ± 0.01 mm against *S. aureus* and *B. subtilis,* respectively, at higher doses only. The Gram-negative bacteria *E. coli* was inhibited up to 17.9 ± 0.03 mm and *K. pneumonia* to an extent of 17.3 ± 0.02 mm zones of inhibition by the EtOAc fraction. Our results agree with the reported data published by Ozkul et al. [28] and Yilmaz et al. [29], who tested different fractions of *Scutellaria* species against selected bacterial and fungal strains. Mostly, Gram-negative strains exhibited high drug resistance in comparison to the Gram-positive bacterial strains, as they do not have high contents of peptidoglycan in their cell walls [30]. The selected plant was found inactive against the fungal strains used, which negate the published findings [28].

### 2.4. Antifungal Activities

The prepared extracts were further tested against *C. Albicans* and *F. oxysporum*, but no activity was observed against these fungal strains.

### 2.5. Antioxidant Activities

**Figure 1:**
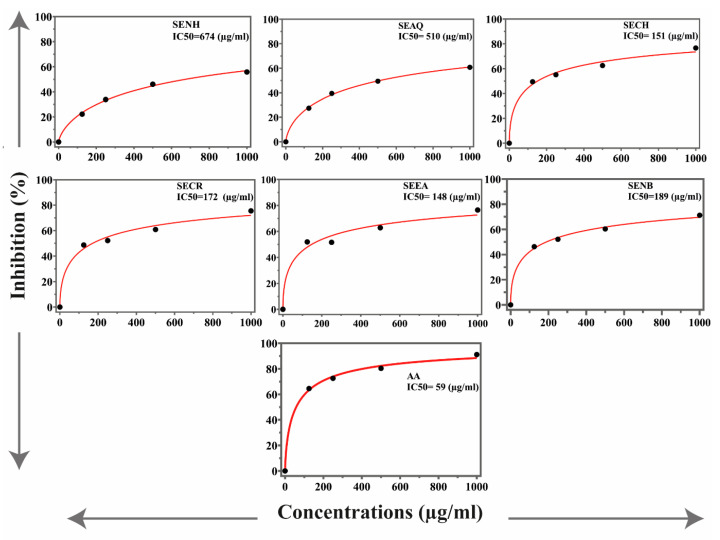
The antioxidant activities of *S. edelbergii* extracts. SE, *S. edelbergii;* NH, n-hexane; AQ, aqueous; CH, chloroform; NB, n-BuOH; EA, EtOAc; CR, crude; AA, ascorbic acid. Data were taken as mean ± SEM and n = 3 with *p* ≤ 0.01.

**Figure 2:**
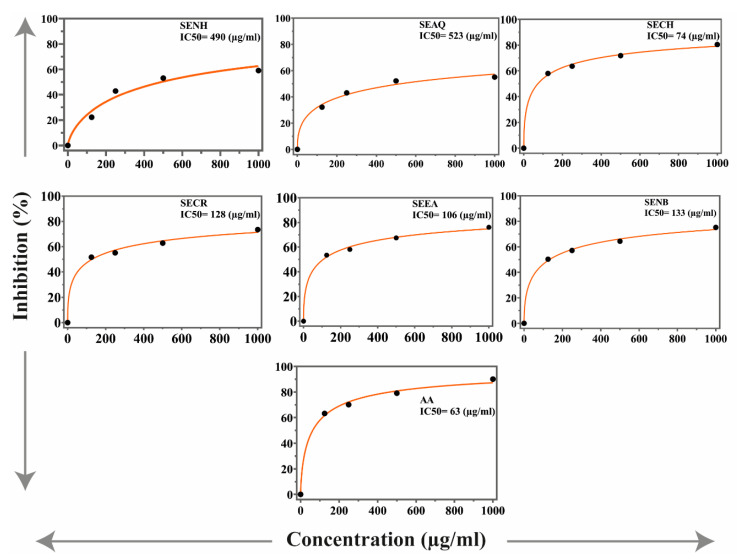
ABTS activities of *S. edelbergii* extracts. SE, *S. edelbergii;* NH, n-hexane; AQ, aqueous; CH, chloroform; NB, n-BuOH; EA, EtOAc; CR, crude; AA, ascorbic acid. Data were taken as mean ± SEM and n = 3 with *p* ≤ 0.01.

The antioxidant potential of the *S. edelbergii* extract and fraction was determined through synthetic free radical DPPH and ABTS assays. The results are presented in Figure 1 and Figure 2. The chloroform fraction exhibited the highest antioxidant activities, with IC_50_ values of 151 and 74 µg/mL in DPPH and ABTS, respectively. However, the EtOAc fraction, crude extract and n-BuOH fraction showed antioxidant activities with value IC_50_ = 148, 172 and 189 µg/mL in DPPH assay, respectively. Similarly, fractions of chloroform, EtOAc, crude extract and n-BuOH fraction demonstrated good activities with IC_50_ values of 106, 128, 133 and 190 µg/mL in ABTS assay, respectively. Meanwhile, aqueous and n-hexane fractions were found least active in both DPPH and ABTS assays. Ascorbic acid was used as a standard that possessed IC_50_ 59 and 63 µg/mL DPPH and ABTS assays, respectively.

*S. edelbergii* was also tested for in vitro antioxidant potentials using different concentrations of the extracts (1000, 500, 250 and 125 µg/mL) using DPPH and ABTS assays and appreciable potential was exhibited in comparison to ascorbic acid used as standard (IC_50_ = 25 µg/mL). Among the investigated fractions against DPPH and ABTS, chloroform fraction with IC_50_ value of 175 and 155 µg/mL was the most potent, followed by EtOAc with IC_50_ values of 205 and 160 µg/mL. Our results match with reported data of Vergun et al. [31], who studied *Scutellaria baicalensis* in the same fractions and concentrations against DPPH radical. Our study is also in agreement with the study of Grzegorczyk-Karolak et al. [32], Delazar et al. [33] and Zengin et al., [34] who have performed the same tests for *S. altissima* and *S. pinnatifida*, *S. Orientalis L*. and *S. salviifolia*. Plants enriched with total phenols and flavonoids are the key source to neutralize the reactive oxygen species; thus, our findings are like the literature available for *S. altissima* and *S. galericulata* [28].

### 2.6. Anti-Inflammatory Activities

The anti-inflammatory activity of *S. edelbergii* crude extract and subfractions are presented in Table 4. Among the tested extracts, the EtOAc fraction was highly potent (54% inhibition) as compared to other extracts. Crude and n-BuOH fraction also exhibited 50% and 47% inhibition, respectively. However, the least activity was obtained for the aqueous fraction (35% inhibition). Diclofenac sodium was used as a standard that produced 74% inhibition of inflammation caused by carrageenan in the tested experimental animals.

The in vivo anti-inflammatory potential of extracts was examined in Swiss albino mice, using the carrageenan-induced assay. The EtOAc fraction was found to be the most active fraction with 47% and 54% inhibition at a concentration of 50 and 100 mg/kg body weight doses followed by chloroform fraction with 42% and 50% inhibition for low and high doses, respectively. The standard diclofenac sodium exhibited 74% inhibition. Our findings agree with the finding of Lee et al. [35], Kim et al. [36] and Liu et al. [26], who investigated *S. baicalensis* and *S. barbata* using similar approach and fractions.

### 2.7. Analgesic Activity

The analgesic activity of *S. edelbergii* extract and sub-fractions in Swiss albino mice are presented in Table 5. The EtOAc fraction exhibited the highest analgesic activity with 55% inhibition, followed by chloroform and n-BuOH fractions with 48% and 42% inhibition, respectively. However, the aqueous fraction demonstrated the least activity. Aspirin was used as a standard which exhibited 68% inhibition of writhes caused by acetic acid.

The analgesic potential of *S. edelbergii* in the form of crude extract and subfraction was determined in Swiss albino mice using different concentrations to cure writhes induced by acetic acid. Aspirin was used as a standard. EtOAc fraction was found to be the most active fraction that exhibited 37% and 55% inhibition at doses of 50 and 100 mg/kg body weight followed by chloroform fraction which brought about 29% and 48% inhibition at doses of 50 and 100 mg/kg body weight, respectively. Our data are in complete agreement with the reported data of Lee et al. [35] and Yimam et al. [37], who have studied *S. baicalensis* using the same assay and fractions.

### 2.8. Isolation of Known Compounds

**Figure 3:**
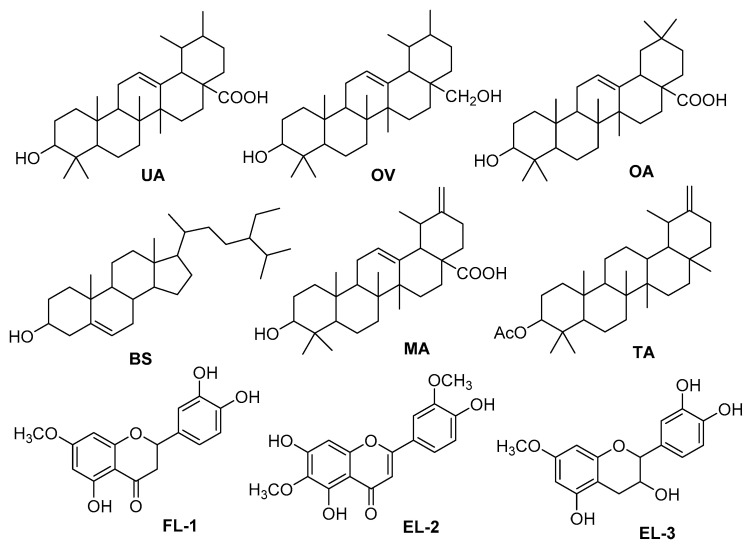
Compounds isolated from *S. edelbergii*.

Six triterpenoids including ursolic acid (**UA**), ovaul (**OV**), oleanolic acid (**OA**), β-sitosterol (**BS**), micromeric acid (**MA**), taraxasterol acetate (**TA**) and three flavonoids viz., 5,3′,4′-trihydroxy-7-methoxy flavone (**FL-1**), 5,7,4′-trihydroxy-6,3′-dimiethoxyflavone (**FL-2**) and 7-methoxy catechin (**FL-3**) were isolated from the methanolic extract of *S. edelbergii* (Figure 3). Their structures were confirmed by combined spectroscopic techniques including 1D (^1^H and ^13^C) and 2D (HMBC, HSQC, COSY) nuclear magnetic resonance (NMR) and high-resolution electrospray ionization-mass spectrometry (ESI-HRMS) and comparison of the spectral data of known compounds with those reported in literature.

Compounds **UA** and **OA** and epimer **MA** have already been reported from the roots and leaves of *S. strigillosa* of the same genus [38,39], while compound **BS** was isolated from the root of *S. baicalensis* [18] and *S. columnae* [40] of the same genus. Several flavonoids, flavanones, flavones and their glycosides were reported from the different species of the same genus [18,38,41]. Altinier et al. [42] reported **UA**, **OA** and **MA** as the main anti-inflammatory principles of the leaves of *Rosmainrus officinalis*. Compounds **MA, TA** and **OV** were not only isolated, for the first time, from the *S. edelbergii* but also from the genus as well, which was previously reported from different plant species [42,43,44,45,46]. The anti-inflammatory and analgesic agent of EtOAc may be due to the presence of these triterpenoid acids in the fraction.

Flavonoids, especially flavanones and flavan-3-ols, are an important class of natural products belonging to a class of plant secondary metabolites having a polyphenolic structure, widely found in fruits, plants, marine species, vegetables and certain beverages [47,48]. They are associated with a broad spectrum of health-promoting effects concerned with a variety of nutraceutical, pharmaceutical, medicinal and cosmetic applications [49]. This is because of their antioxidative, anti-inflammatory, anti-mutagenic and anti-carcinogenic properties coupled with their capacity to modulate key cellular enzyme functions [47]. The antioxidant activity (DPPH and ABTS) of the chloroform fraction could be possible due to the presence of these flavonoids.

The plant and its derived products are varying under environmental gradients such as water, temperature and light [50]. Mainly, the genus is a rich basis of flavonoid contents which are mainly influenced by the optimum level of the environmental gradient for the genus *Scutellaria;* thus, our finding agrees with the reported data of Chen et al. [51], who studied *S. baicalensis*. As mostly the species of genus *Scutellaria* found with their habitats on the mountain tail with colder dry weather, similarly, the understudy sample was collected from mountain tail enriched with flavonoids and phenols. Thus, our results are matching with the findings of Grzegorczyk-Karolak et al. [32], who investigated *S. altissima* and *S. galericulata* for mentioned studies. It is revealed from *S. altissima* having high contents of phenols and flavonoids observed for *S. edelbergii* content [52]. Moreover, the bioactive compound scutellarin is present in the *Scutellaria* beside its habitats and environment, detected by Matsa et al. [53].

## 3. Material and Methods

### 3.1. General Instrumentation

The ^1^H- and ^13^C NMR spectra were measured on 600 MHz and 150 MHz NMR spectrometer (BRUKER, Zürich, Switzerland) using the solvent peaks (CDCl_3_, δ_H_: 7.26; δ_C_: 77.0), (CD_3_OD, δ_H_: 4.87; δ_C_: 48.5) as internal references. Data were determined in the following order: chemical shift (δ) in ppm; multiplicities are indicated s = singlet, d = doublet, t = triplet, dd = doublet of doublet, m = multiplet; coupling constants (J) are in hertz (Hz). High-resolution electrospray ionization mass spectrometry (HR-ESI-MS) spectra were recorded on Agilent 6530 LC Q-TOF (country of origin USA/EU, made in Singapore). Column chromatography was carried out by using silica gel of the selected particle size of 100–200 mesh. For TLC, pre-coated aluminum sheets (TLC silica gel 60 F254, Merck, Darmstadt, Germany) were used. Infrared (IR) spectra were recorded on a Bruker ATR-Tensor 37 spectrophotometer, Bruker (Ettlingen, Baden-Württemberg, Germany) with wave numbers (ν) in cm^−1^. Visualization was accomplished with UV light (254 and 366 nm) or I_2_ stain and by spraying with the ceric sulfate (Ce(SO_4_)_2_) reagent.

### 3.2. Plant Collection and Identification

The whole plant material of *S. edelbergii* was collected (April–June 2019) from Kalam, District Swat, Khyber Pakhtunkhwa, Pakistan, and identified by the plant taxonomist Prof. Mehboob Rahman, Matta College Swat, KPK, Pakistan. The plant specimen (AWKUM/Herb/2234) was placed in the herbarium of Abdul Wali Khan University, Mardan.

*S. edelbergii* is a perennial herb, spreading with a thick woody rootstock. Stems are very slender, procumbent or weakly ascending, round-quadrangular, leafy and much-branched. Leaves are thick-textured, triangular or narrow ovate and acute. Flowers subtended by elliptic to broad elliptic bracts which are entire, cuneate to acute, Calyx with a small often purple scutellum, enlarging in fruit to 3 mm with a 2.5 mm high scutellum. Petals are yellow or blue violet, with the darker lower lip spreading erect or erect, densely glandular pilose. Flowering is produced in April to July found between 1660–2200 m. Furthermore, it grows in moist, loamy soil, moist temperate regions and spread in different areas of northwest Pakistan, mainly influenced by the tail-end of monsoon conditions.

### 3.3. Extraction and Fractionation

**Figure 4:**
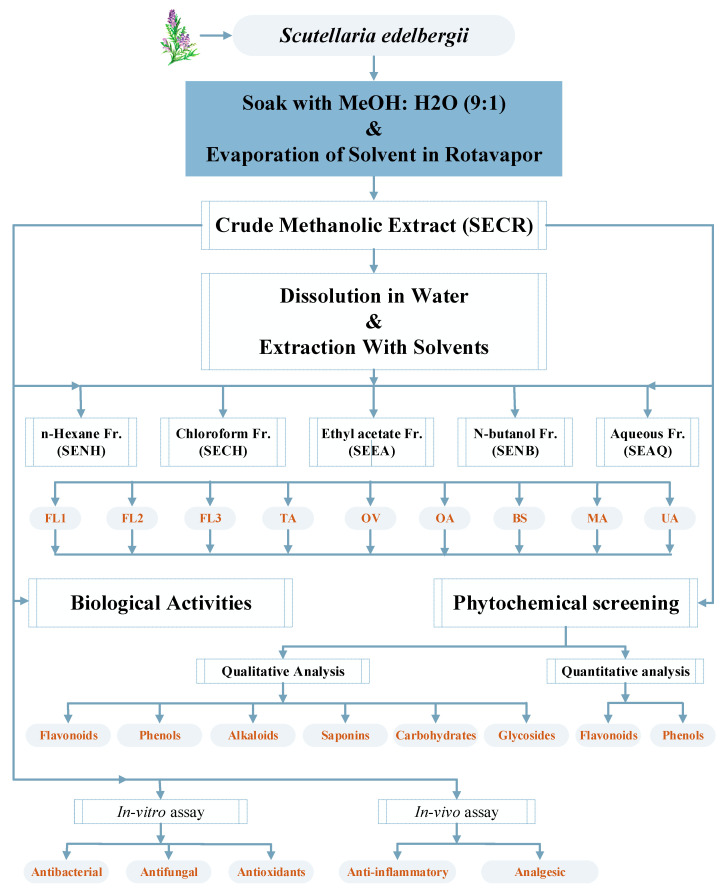
Schematic representation of the current project.

The whole air-dried plant material of S. *edelbergii* was washed with tap water to remove soil/silicate particles and dried at room temperature for three weeks under shade to avoid loss of volatile components. The dried plant material was crushed into very fine powder through an electric grinder. The powders were kept in the refrigerator at 4 °C until further use. To prepare the crude extract, 2 kg of fine powder were soaked in commercial-grade methanol (6 L). The mixture was periodically shaken and then filtered through a muslin cloth after 21 days. The residue left was again immersed in 10% water/methanol for additional 21 days and filtered again. The filtrates from both the steps were combined and subjected to evaporation in a rotary evaporator at 40 °C. The semi-solid mass obtained was then placed in the open air till dryness; later, 600 g of crude extract were obtained and stored in airtight containers for further use. To prepare different fractions, 500 gm of crude extract were immersed in 1 L distilled water, which was then subjected to solvent-solvent extraction in a separating funnel from less to high polar solvents (n-hexane < chloroform < ethyl acetate < n-butanol) (Figure 4). The obtained fractions were evaporated in a rotary evaporator at 40 °C that yielded the dry mass of 21, 19, 20 and 18 g of n-hexane, chloroform, EtOAc and n-BuOH, respectively. In comparison, the aqueous extract was obtained in more quantity (35 g).

The obtained EtOAc fraction was further subjected over the silica gel (70–230 mesh; Merck) column chromatography (CC) using *n*-hexane, *n*-hexane/ethyl acetate and ethyl acetate/MeOH with 10% increasing polarity to afford twelve fractions (SE_1_–SE_12_). After taking TLC, four factions (SE_3_–SE_6_; 10–20% *n*-hexane/ethyl acetate) were combined and subjected to further small CC using *n*-hexane/ethyl acetate with increasing polarity (1:9, 2:8, 4:6, 6:4) to afford four sub-fractions (SSE_1_–SSE_4_). SSE_2_ (10–20% *n*-hexane/ethyl acetate) was further subjected to CC and preparative TLC to afford known compounds **BS** (18.5 mg), **UA** (22.0 mg), **OV** (5.0 mg), **MA** (7.0 mg) and **OA** (6.5 mg). Similarly, flavonoids **FL-1** (4.5 mg), **FL-3** (6.0 mg) and **FL-2** (5.0 mg) were isolated from the chloroform fraction of S. *edelbergii* using solvent system of 40–60% *n*-hexane/ethyl acetate (Figure 4). All the isolated compounds are known (isolated first time from the source) and characterized by detail spectroscopic techniques. The detail NMR data, physical state and mass spectrometry are given below (show in Supplementary Materials).

(3β)-3-hydroxy-urs-12-en-28-oic acid (**UA**): Colorless crystals; ^1^H-NMR (600 MHz, Chloroform-d, *δ* = ppm): 5.23 (1H, br. s), 3.20 (1H, t, 10.8, 6.6 Hz), 2.17 (1H, d, 10.8 Hz), 1.29 (3H, s), 1.11 (3H, s), 0.99 (3H, s), 0.90 (3H, s), 0.87 (3H, s), 0.86 (3H, s), 0.69 (3H, s), ^13^C-NMR (150 MHz, Chloroform-d, *δ* = ppm): 181.2, 137.9, 125.9, 79.0, 55.2, 52.7, 47.9, 47.6,42.0, 39.5, 39.1, 38.8, 38.7, 38.6, 37.0, 36.7, 33.0, 30.6, 29.7, 28.1, 28.0, 27.2, 24.2, 23.6, 23.3, 21.2, 18.3, 17.1, 17.0, 15.6, 15.5; ESI-HRMS (negative): *m*/*z* 455.3531 [M − H]^+^ [50,54].

Ovaul (**OV**): ^1^H-NMR (600 MHz, Chloroform-d): White powders; 5.11 (1H, t, *J* = 3.6, H-12), 3.51 (1H, d, *J* = 10.8, H-28a), 3.20 (1H, dd, *J* = 11.4, 4.8, H-3α), 3.19 (1H, d, *J* = 10.8, H-28b), 1.11, 1.00, 0.95, 0.93, 0.79 (each 3H, s), 0.94, 0.81 (each 3H, d, *J* = 6.0).^13^C NMR (150 MHz, Chloroform-d, δ = ppm): 138.6, 125.0, 79.0, 69.9, 55.1, 54.0, 47.6, 42.0, 40.0, 39.4, 39.3, 38.7, 38.0, 36.8, 35.1, 32.8, 30.6, 28.1, 27.2, 25.9, 23.4, 23.3, 23.3, 21.3, 18.3, 17.3, 16.7, 15.6, 15.6; ESI-HRMS (positive): *m*/*z* 443.3311 [M + H]^+^ [50].

3β-hydroxy-olea-12-en-28-oic acid (**OA**): White crystals; ^1^H-NMR (600 MHz, Chloroform-d): 5.28 (1H, br. s), 3.23 (1H, s, dd, 11.4, 3.6 Hz), 2.83 (1H, dd, 13.8, 3.6 Hz), 1.13 (3H, s), 0.97 (3H, s), 0.94 (3H, 0.94 (3H, s), 0.88 (3H, s), 0.85–0.75 (6H, s); ^13^C NMR (150 MHz, Chloroform-d, δ = ppm): 182.9, 143.6, 122.6, 79.0, 55.2, 47.6, 46.5, 45.9, 41.6, 41.0, 39.3, 38.7, 38.4, 37.1, 33.8, 33.1, 32.6, 32.4, 30.7, 28.1, 27.7, 27.2, 25.9, 23.6, 23.4, 22.9, 18.3, 17.1, 15.5, 15.3; ESI-HRMS (negative): *m*/*z* 455.3516 [M − H]^+^ [54].

β-Sitosterol (**BS**): White powder; ^1^H-NMR (600 MHz, Chloroform-d): 5.33 (1H, br. s), 3.51 (1H, br. t), 1.03 (S, 3H), 0.93 (3H, d, J = 6.5 Hz), 0.84 (3H, t, J = 7.2 Hz), 0.83 (3H, d, J = 6.4 Hz), 0.81 (3H, d, J = 6.4 Hz), 0.68 (3H, s); ^13^C NMR (150 MHz, Chloroform-d, *δ* = ppm): 140.8, 121.7, 71.8, 56.8, 56.1, 50.1, 45.9, 42.3, 39.8, 37.3, 36.5, 36.1, 34.0, 31.9, 31.8, 29.7, 29.2, 28.2, 26.1, 24.3, 23.1, 21.1, 19.8, 19.4, 19.0, 18.8, 12.0, 11.8; ESI-HRMS (positive): *m*/*z* 437.3417 [M + Na]^+^ [55].

Micromeric acid (**MA**): Colorless powder; ^1^H NMR (600 MHz, Pyr-d, *δ* = ppm): 5.52 (td, *J* = 6.2, 1.0 Hz, 1H), 4.83 (dd, *J* = 13.7, 1.1 Hz, 1H), 4.78 (dd, *J* = 13.7, 1.1 Hz, 1H), 3.49 (td, *J* = 7.1, 5.0 Hz, 1H), 2.79 (pd, *J* = 7.4, 6.8, 1.3 Hz, 1H), 2.67–2.65 (m, 2H), 2.35–2.29(m, 2H), 2.15–2.09 (m, 3H), 1.99–1.77 (m, 4H), 1.61–1.36 (m, 4H), 1.34–1.22 (m, 2H), 1.25–1.16 (m, 2H), 1.20–1.11 (m, 2H), 1.14 (s, 3H), 1.12 (s, 2H), 1.00–0.95 (s, 9H), 0.92 (s, 3H), 0.86 (s, 3H); ^13^C-NMR (150 MHz, Pyr-d, *δ* = ppm): 180.4, 154.3, 139.8, 126.2, 105.6, 78.6, 56.4, 54.1, 48.6, 48.5, 43.0, 40.5, 39.6, 39.9, 39.6, 38.02, 37.86, 34.15, 31.64, 29.38, 29.2, 28.7, 24.2, 24.2, 21.9, 19.3, 18.0, 18.0, 17.1, 16.2; ESI-HRMS (positive): *m*/*z* 455.3563 [M + H]^+^ [46,47].

Taraxasterol acetate (**TA**): White powder; ^1^H NMR (600 MHz, Chloroform-d, *δ* = ppm) 4.59 (dt, *J* = 10.1, 2.4 Hz, 2H), 4.46 (dd, *J* = 10.9, 5.6 Hz, 1H), 2.11–2.00 (m, 1H), 2.02 (s, 3H), 1.74–1.45 (m, 4H), 1.36 (s, 2H), 1.43–1.29 (m, 3H), 1.30–1.17 (m, 2H), 1.15–1.03 (m, 1H), 1.00 (t, *J* = 3.4 Hz, 5H), 0.98–0.90 (m, 3H), 0.91 (s, 3H), 0.87–0.80 (m, 9H), 0.82 (s, 3H); ^13^C NMR (150 MHz, Chloroform-d, *δ* = ppm) 171.01, 154.64, 107.10, 80.98, 55.45, 55.44, 50.40, 48.65, 42.03, 40.92, 39.38, 39.16, 38.86, 38.44, 38.29, 37.80, 37.05, 34.53, 33.99, 27.94, 26.64, 26.15, 25.61, 25.48, 23.69, 21.46, 21.32, 19.48, 18.18, 16.49, 16.33, 15.89, 14.72; ESI-HRMS (positive): *m*/*z* 491.3731 [M + Na]^+^ [48,49].

5,3′,4′-trihydroxy-7-methoxy flavone (**FL-1**): Yellow powder; ^1^H-NMR (600 MHz, DMSO-d6) δ 7.53 (1H, br. s, H-2′), 7.48 (lH, d, J = 8.6, H-6′), 6.87 (1H, d, J = 8.4, H-5′), 5.98 (2H, d, J = 2.0, Hz, H-6 and H-8), 5.22 (lH, dd, J = 12.2, 3.2 Hz, H-2), 3.82 (3H, s, OMe), 2.90 (2H, m, H-3); ^13^C-NMR (150 MHz, DMSO-d, *δ* = ppm): 195.8, 167.0, 166.6, 164.2, 149.1, 143.9, 129.4, 123.0, 115.7, 114.6, 113.5, 96.7, 95.6, 79.0, 51.7, 43.2; ESI-HRMS (positive): *m*/*z* 289.0706 [M − CH_2_ + H]^+^ [56,57,58,59].

5,7,4′-trihydroxy-6,3′-dimiethoxyflavone (**FL-2**); Yellow powder; ^1^H-NMR (600 MHz, CH_3_OH-d, *δ* = ppm): 7.43 (1H, dd, J = 8.4, 1.8 Hz, H-6′), 7.39 (1H, d, J = 1.8 Hz, H-2′), 6.90 (1H, d, J = 8.4 Hz, H-5′), 6.79 (1H, s, H-8), 6.60 (1H, s, H-3), 3.97 (3H, s, OCH_3_), 3.82 (3H, s, OCH_3_); ^13^C-NMR (150 MHz, *CH_3_OH*-d, *δ* = ppm): 184.2, 166.8, 160.6, 154.9, 151.3, 149.1, 147.1, 133.2, 123.5, 120.4, 116.7, 114.1, 106.5, 103.8, 92.2, 61.0, 56.9; ESI-MS (positive): *m*/*z* 352.94 [M + Na]^+^ [60,61,62].

7-methoxy catechin (**FL-3**): Yellow powder, brown gummy solid; ^1^H-NMR (600 MHz, DMSO-d, *δ* = ppm): 6.70 (d, 1H, J = 11.2 Hz, H-5′), 6.59 (dd, 1H, J = 10.8 & 3.2 Hz, H-6′), 6.59 (d, 1H, J = 3.2 Hz, H-2′), 5.86 (s, 1H, H-8), 5.69 (1H, s, H-6), 4.47 (d, 1H, J = 10.4 Hz, H-2), 3.71 (d, J = 6.8, 10.4 & 11.6 Hz, H-3), 3.16 (s, OCH_3_, H-11), 2.54 (dd, 1H, J = 20.8 and 6.8 Hz, H-4α), 2.50 (1H, m, H-4β); ^13^C-NMR (150 MHz, DMSO-d, *δ* = ppm): 156.3, 156.1, 155.2, 144.8, 130.5, 118.4, 115.1, 114.4, 99.1, 95.2, 93.9, 80.8, 66.2, 48.6, 27.6; ESI-HRMS (positive): *m*/*z* 291.0729 [M − CH_2_ + H] ^+^ [63,64,65].

### 3.4. Preliminary Phytochemical Analysis

Crude extract and their fractions were subjected to preliminary phytochemical tests to confirm the presence of flavonoids, phenols, alkaloids, saponins, glycosides and carbohydrates using reported methods [66,67,68]. The crude extract along with different fractions (1 g) were dissolved in the 5 mL DMSO (99.99%, Fischer Scientific, Loughborough, UK) to make a stock solution, and DMSO was further used for re-solubilization (Figure 4).

#### 3.4.1. Flavonoids

A few drops from the stock solution of crude extract and different fractions of *S. edelbergii* were mixed with diluted in 5% NaOH solution, followed by the addition of a few drops of hydrochloric acid (HCl). The presence of flavonoids was detected through color changes from yellow to colorless.

#### 3.4.2. Phenols

A few drops from the stock solution of crude extract and different fractions were added to the FeCl_3_ solution, followed by vigorous shaking. The appearance of a bluish-green color confirms the presence of phenols.

#### 3.4.3. Alkaloids

Qualitative detection of alkaloids was performed by mixing 0.5 mL of stock solution of crude extract and fractions with 2% H_2_SO_4_ solution, followed by heating for 3 min. Then, some drops of Dragendorff’s reagent were added to the mixture and allowed to cool. The appearance of an orange-red color precipitate revealed the presence of alkaloids.

#### 3.4.4. Saponins

From the stock solution of *S. edelbergii* extracts and fractions, a few drops were mixed with 0.4 mL of distilled water, followed by shaking. After 5 min, the mixture was boiled and resulted in the formation of small bubbles (froth), which confirmed the presence of saponins.

#### 3.4.5. Carbohydrates

The presence of carbohydrates was determined by mixing 3 mL of extract solution with 2 mL of research-grade Benedict’s reagent. The mixture was then placed in a hot water bath for 3 min. The appearance of a reddish-brown precipitate revealed the presence of carbohydrates.

#### 3.4.6. Glycosides

A few drops of HCl were mixed with small quantities of extract solutions that caused hydrolysis of glycosides, which were then neutralized through the addition of (how much amount) NaOH solution. After neutralization, about 0.5 mL from Fehling’s A and B solutions were added to each extract mixture. Glycoside’s confirmation was revealed by the appearance of a red color precipitate.

#### 3.4.7. Flavonoids and Phenolic Contents

The selected plant was investigated for quantitative estimation of the total phenolic and flavonoids contents in crude extract and subfraction using standard procedure [69].

#### 3.4.8. Estimation of Total Phenolic Contents

The phenolic content in *S. edelbergii* extracts and subfractions was determined using the Folin–Ciocalteu reagent. The total phenolic content was measured as milligrams of gallic acid equivalent (mg GAE/g) per gram of dry sample mass. Different extracts in distilled methanol (5 mg in 5 mL) were mixed with 10 mL distilled water and placed undisturbed for 5 min. About 1 mL from each mixture was taken in a test tube, and its volume was raised to 10 mL by adding distilled water. Then, 1 mL of Folin-Ciocalteu reagent was added to each test tube and incubated for 6 min. After incubation, 10 mL of 7% sodium carbonate solution were added. The final volume of the reaction mixture was raised to 26 mL through the addition of distilled water and incubated for 90 min at room temperature. The absorbance was noted at 760 nm using a UV-visible spectrophotometer.

#### 3.4.9. Quantification of Total Flavonoid Contents

The total flavonoid content was represented as a quercetin equivalent (mg QE/g) of the dry sample mass of the extract. About 9 mL of the distal water was mixed with 1 mL of each extract stock solution, along with 1 mL of 5% NaNO_2_ in a test tube, which was then incubated for 6 min. Then, 2 mL of 10% AlCl_3_ was added to each test tube, and the reaction mixture was then placed undisturbed for 5 min. Finally, 2 mL of 1 M sodium hydroxide was added to each test tube, and the absorbance of the resultant mixture was noted at 510 nm via a UV-visible spectrophotometer.

### 3.5. In Vitro Biological Activities

In vitro antibacterial and antifungal activities were carried out on *E. coli*, *K. pneumoniae*, *B. Subtilis*, *S. aureus*, *C. albican* and *F. oxysporum* strains. The fungal and bacterial strains were obtained from the laboratory of Microbiology, verified by Dr. Hazir Rahman, Chairman, Department of Microbiology, AWKUM, Mardan.

#### 3.5.1. Antibacterial Activities

The crude extract and different fractions (1 g) were dissolved in the 5 mL DMSO to make a stock solution and DMSO was further used for re-solubilization for antibacterial activity. The antimicrobial potential of the crude extract and subfractions of *S. edelbergii* was examined through agar well diffusion assay [70]. Twenty-eight grams (28 g) of nutrient agar were added to 1000 mL of distilled water and shaken vigorously until its complete dissolution. The nutrient agar media, loop, corn borer and Petri dishes were autoclaved at 121 °C for 15 min. In an aseptic condition of laminar flow hood, 20 mL agar were poured to each Petri plate and allowed to solidify. Bacterial strains (*E. coli, K. pneumonia*, *S. aureus, B. subtilis*) were inoculated through a wire loop using a safety kit using concentration of bacterial cell density of 1.5 × 10^8^ CFU/mL. Four wells of 3 mm size were made on a cork borer in each plate with the same distances. The selected plant extract at concentrations of C_1_ = 50 µL and C_2_ = 100 µL were poured into the wells. The same method was used for positive control. Levofloxacin and Erythromycin were used as a positive control for Gram-positive and Gram-negative strains, respectively. Dimethyl sulfoxide (DMSO) was used as a negative control. The Petri plates were placed in the incubator at 37 °C for 24 h. Three biological replicates with 2 different volumes were used. Later, plates were then taken out to measure the zone of inhibition around each hole. The results were taken in triplicate.

#### 3.5.2. Antifungal Activity

For antifungal assay, the crude extract and different fractions (1 g) were dissolved in the 5 mL DMSO to make a stock solution and DMSO was further used for re-solubilization. Antifungal activities were performed on potato dextrose agar media [70]. Thirty-nine grams (39 g) of potato dextrose agar media were added to 1 L distilled water and sterilized in an autoclave at 121 °C. The media plates were incubated for 24 h in an incubator to avoid contamination of unwanted strains. Then, four holes were created in media under laminar flow and holes were poured with extract solution. The plates inoculated with fungal strains from inoculum having concentration 10^8^–10^9^ CFU/mL and left for 3 days in the incubator at 25 °C. The zone of inhibition of standard and test extracts were measured in mm.

#### 3.5.3. Antioxidant Activity (DPPH and ABTS)

To evaluate the antioxidant potential of crude extract and sub-fractions, 2,2-diphenyl-1-picrylhydrazyl (DPPH) and (2,2′-azino-bis (3-ethylbenzothiazoline-6-sulfonic acid) (ABTS) [71] were used. In the DPPH assay, 3 mg of DDPH was dissolved in 100 mL distilled methanol and kept in the dark for 30 min for the formation of free radicals in the solutions. Different dilutions of each extract (1000, 500, 250, 125 µg/mL) were also prepared. Then, 2 mL of each dilution were added with 2 mL DPPH stock solution and incubated for 20 min in the dark. After incubation, the absorbance of each sample was measured at 517 nm by UV/Vis spectrophotometer. Similar dilutions of ascorbic acid were also prepared and incubated with 2 mL of DPPH stock solution for the aforementioned duration to determine its antioxidant potential. The following formula was used to determine the antioxidant potentials of the samples:

(1) $$\%\;Scavenging\;activity=\frac{A-B}{A}\times 100$$

where *A* is the control absorbance and *B* is the standard absorbance

The antioxidant potential of these extracts was also determined by the ABTS method. A total of 383 mg of ABTS and 66.2 mg of Potassium persulfate (K_2_S_2_O_8_) were dissolved individually in 100 mL methanol and mixed. Later, 2 mL of the mixture were incubated with 2 mL of extract dilutions for 25 min. The absorbance was noted at 746 nm by UV spectrophotometer. (The free radical scavenging potential of the extracts was calculated by Equation (1)).

### 3.6. In Vivo Pharmacological Activities

For in vivo anti-inflammatory and analgesic activities, approval was taken from the ethical committee (No: AWKUM/Bot/2019/1720, Date: 29 January 2019) of Abdul Wali Khan University Mardan, Mardan (AWKUM) (provided in hard to the publisher).

#### 3.6.1. Experimental Animals 

Healthy Swiss albino mice (approved by the ethical committee (No: AWKUM/Bot/2019/1720, Date: 29 January 2019), Botany Department, AWKUM) of either sex was placed in rubber cages in germ-free conditions for 45 days at around 20 °C and fed with rodent pellet food and water.

#### 3.6.2. Anti-Inflammatory Activities

Twenty (20) Swiss albino mice with a bodyweight of 24–30 g were used in this study. The mice were brought from the Veterinary Center Peshawar and kept in the animal house of AWKUM. The animals were given nutrient pellets and water. ARRIVE guidelines were followed while selecting sample size, experimental design and inclusion and exclusion techniques for statistically acceptable/satisfactory results [72].

The animals were divided into four groups as per the given details (five mice in each group):

Group A: 1 mL carrageenan was injected to induce paw edema in mice.

Group B: 1 mL carrageenan solution and Diclofenac sodium (as a standard drug, 50 mg/kg body weight) were injected.

Group C: 1 mL carrageenan was injected with 50 mg/kg body weight of each extract.

Group D: 1 mL carrageenan was injected with 100 mg/kg body weight of each extract.

The diameter of the carrageenan injected paw edema was measured after 1, 2 and 3 h. The average % inhibition was calculated in comparison with the standard by the following formula:

(2) $$\%\;inhibition=\frac{A-B}{A}\times 100$$

where *A* is the induced paw diameter and *B* is the tested drug (in case of analgesic activities, *A* is writhes induced by acetic acid).

#### 3.6.3. Analgesic Activities

The analgesic activity was also determined in Swiss albino mice. The animals were divided into four groups (5 mice each) as per the given details:

Group A: 1 mL acetic acid solution was injected into this group to cause writhing.

Group B: 1 mL acetic acid and 1 mL aspirin (standard drug) were injected.

Group C: 1 mL acetic acid and 50 mg/kg body weight of each extract were injected.

Group D: 1 mL acetic acid and 100 mg/kg body weight of each extract were injected.

After injection, the rate of writhing was noted for 10 min, and % inhibition was noted by comparing it with the standard by Equation (2).

### 3.7. Statistical Analysis

All the data were taken in triplicate and analyzed through one-way ANOVA (analysis of variance) followed by Bonferroni’s test to determine significance level [*p* = (≤0.05) and *p* = (≤0.01)] of all the obtained results. A nonlinear regression graph was constructed between % inhibition and concentration, and the IC_50_ was measured with the GraphPad Prism 9 program for windows (GraphPad-Software, San Diego, California, 2020) using Equation (1).

Y=100/1+(^HillSlope)

where 1 = the concentration of the inhibitors used, *Y* = the inhibitor’s reaction and *HillSlope* indicates the steepness of the curves.

## 4. Conclusions

The present analysis revealed that *S. edelbergii* is a rich source of secondary metabolites that could be effectively used as a remedy for several health complications. The EtOAc fraction exhibited the highest phenolic and flavonoid contents. Significant in vitro antibacterial activity was observed, especially against Gram-positive bacterial strains in comparison to the Gram-negative strain used in the study. The tested extracts were found inactive against the selected fungal strains. The chloroform fraction was the most effective scavenger of the DPPH and ABTS free radicals. The EtOAc fraction was found to act as an anti-inflammatory and analgesic agent in Swiss albino mice. Furthermore, six known triterpenoids including **UA**, **OV**, **OA**, **BS**, **MA** and **TA** as well as three flavonoids viz., **FL-1**, **FL-2** and **FL-3** were isolated, for the first time, from the methanolic extract of *S. edelbergii*. Based on the results of our investigation, the selected plant could be used as an antibacterial, analgesic, anti-inflammatory and antioxidant agent due to the presence of triterpenoids and flavonoids. However, further studies are needed to determine more responsible compounds of the observed biological and pharmacological activities.

## Figures and Tables

**Table 1:** Phytochemical composition of *S. edelbergii* extracts.

Fractions	Flavonoids	Phenols	Alkaloids	Saponins	Carbohydrates	Glycosides
SENH	-	+	-	-	-	-
SEAQ	+	+	+	+	+	+
SECH	+	+	+	-	-	-
SENB	+	+	+	+	+	+
SEEA	+	+	+	+	+	+
SECR	+	+	+	+	+	+

SE, *S. edelbergii*; NH, n-hexane; AQ, aqueous; CH, chloroform; NB, n-BuOH; EA, EtOAc; CR, crude. The + indicates the presence of phytochemical group.

**Table 2:** Total phenols and flavonoids estimation of *S. edelbergii* extracts.

Fractions	Total Phenolic Contents (mg GAE/g) Dry Fractions Mean ± SEM	Total Flavonoid Contents (mg QE/mg) of Dry Fractions Mean ± SEM
SENH	36.5 ± 0.11 **	41.0 ± 0.29 **
SEAQ	49.3 ± 0.11 **	52.5 ± 0.37 **
SECH	58.1 ± 0.14 **	64.0 ± 0.37 **
SENB	64.5 ± 0.17 **	75.4 ± 0.28 **
SEEA	79.2 ± 0.30 **	84.0 ± 0.39 **
SECR	69.9 ± 0.13 **	80.5 ± 0.42 **

SE, *S. edelbergii*; NH, n-hexane; AQ, aqueous; CH, chloroform; NB, n-BuOH; EA, EtOAc; CR, crude; TPC, total phenolic contents; TFC, total flavonoid contents; QE, quercetin equivalent; GAE, gallic acid equivalent; n, 3. Data are expressed as mean ± SEM (standard error mean), *p* ≤ 0.01 ** Mean ± SEM.

**Table 3:** Antibacterial activities of different extracts of *S. edelbergii*.

**Antibacterial Activities of Extracts and Fractions against Gram-Negative Bacterial Strains**				
**Fractions**	***Escherichia coli***		***Klebsiella pneumonia***	
	C_1_	C_2_	C_1_	C_2_
SECR	12.3 ± 0.02 *	16.3 ± 0.03 *	13.6 ± 0.04 *	16.6 ± 0.05 *
SEEA	14.5 ± 0.04 *	17.9 ± 0.03 *	14.3 ± 0.02 *	17.3 ± 0.02 *
SECH	13.6 ± 0.01 *	16.3 ± 0.02 *	13.9 ± 0.05 *	16.6 ± 0.01 *
SENB	13.3 ± 0.03 *	16.6 ± 0.04 *	12.6 ± 0.03 *	16.3 ± 0.04 *
SENH	12.6 ± 0.02 *	15.6 ± 0.01 *	12.3 ± 0.04 *	15.3 ± 0.01 *
SEAQ	12.3 ± 0.01 *	15.3 ± 0.01 *	11.9 ± 0.01 *	14.3 ± 0.02 *
Levofloxacin	17.6 ± 0.03	24.3 ± 0.02	16.3 ± 0.04	21.3 ± 0.04
DMSO	-	-	-	-
**Antibacterial activities of extract and fractions against Gram-positive bacterial strains**				
**Fractions**	***Staphylococcus aureus***		***Bacillus subtilis***	
	C_1_	C_2_	C_1_	C_2_
SECR	14.03 ± 0.03 *	18.03 ± 0.01 *	13.07 ± 0.03 *	18.21 ± 0.03 *
SEEA	15.03 ± 0.01 *	19.07 ± 0.02 *	14.97 ± 0.01 *	18.87 ± 0.04 *
SECH	14.57 ± 0.03 *	17.03 ± 0.03 *	14.07 ± 0.03 *	17.09 ± 0.01 *
SENB	13.87 ± 0.02 *	16.71 ± 0.02 *	14.07 ± 0.04 *	16.27 ± 0.03 *
SENH	13.03 ± 0.04 *	16.03 ± 0.01 *	12.03 ± 0.01 *	15.81 ± 0.01 *
SEAQ	12.03 ± 0.01 *	15.11 ± 0.04 *	11.87 ± 0.02 *	14.51 ± 0.01 *
Erythromycin	17.03 ± 0.03	22.09 ± 0.03	16.90 ± 0.05	22.03 ± 0.04
DMSO	-	-	-	-

SE, *S. edelbergii;* NH, n-hexane; AQ, aqueous, CH, chloroform; NB, n-BuOH; EA, EtOAc; CR, crude; C, concentration; C_1_, 50 µL; C_2_, 100 µL. *p* ≤ 0.05 *; n, 3, compared with positive control (Levofloxacin and Erythromycin) and negative control (dimethyl sulfoxide (DMSO)). Mean ± SEM, n = 3 (data were taken as mean ± SEM). The positive control is Erythromycin and Levofloxacin; the negative control is DMSO.

**Table 4:** Anti-inflammatory potentials of *S. edelbergii* extracts.

		Paw Volume Changes (Mean ± SEM)				
Treatment	Dose (mg/kg)	1 h	2 h	3 h	Average Reading	% Inhibition
Carrageenan	1 mL	1.07 ± 0.03	1.31 ± 0.01	1.63 ± 0.02	1.33 ± 0.02	
Diclofenac	50	0.51 ± 0.02	0.35 ± 0.05	0.19 ± 0.03	0.35 ± 0.05	74
SECR	50	0.91 ± 0.07	0.82 ± 0.01	0.69 ± 0.05	0.80 ± 0.04 *	39
	100	0.79 ± 0.04	0.66 ± 0.02	0.53 ± 0.06	0.66 ± 0.04 *	50
SEEA	50	0.82 ± 0.06	0.73 ± 0.03	0.60 ± 0.01	0.71 ± 0.03 *	47
	100	0.74 ± 0.03	0.61 ± 0.02	0.49 ± 0.03	0.61 ± 0.02 *	54
SECH	50	0.88 ± 0.05	0.79 ± 0.02	0.65 ± 0.04	0.77 ± 0.03 *	42
	100	0.79 ± 0.02	0.68 ± 0.04	0.56 ± 0.01	0.67 ± 0.02 *	50
SENB	50	0.89 ± 0.03	0.78 ± 0.05	0.70 ± 0.02	0.79 ± 0.03 *	41
	100	0.81 ± 0.04	0.70 ± 0.02	0.62 ± 0.01	0.71 ± 0.02 *	47
SENH	50	0.93 ± 0.01	0.83 ± 0.06	0.74 ± 0.03	0.83 ± 0.03 *	38
	100	0.86 ± 0.03	0.77 ± 0.03	0.68 ± 0.02	0.77 ± 0.03 *	42
SEAQ	50	0.95 ± 0.02	0.87 ± 0.02	0.79 ± 0.03	0.87 ± 0.02 *	35
	100	0.88 ± 0.03	0.81 ± 0.02	0.75 ± 0.01	0.81 ± 0.02 *	39

SE, *S. edelbergii;* NH, n-hexane; AQ, aqueous; CH, chloroform; NB, n-BuOH; EA, EtOAc; CR, crude. Diclofenac sodium is the positive control. n = 3 with *p* ≤ 0.05 *; data were taken as mean ± SEM.

**Table 5:** Analgesic activities of *S. edelbergii* fractions.

Treatment	Dose (mg/kg)	No of Writhes Mean ± SEM	% Reduction in Writhing
Acetic acid	1 mL	29.6 ± 0.02	
Aspirin	1 mL	9.3 ± 0.02	68
SECR	50	21.4± 0.07 **	28
	100	17.3 ± 0.04 **	42
SEEA	50	18.6 ± 0.06 **	37
	100	13.3 ± 0.03 **	55
SECH	50	21.2 ± 0.05 **	29
	100	15.3 ± 0.02 **	48
SENB	50	21.3 ± 0.03 **	28
	100	17.3 ± 0.04 **	42
SENH	50	20.3 ± 0.01 **	31
	100	17.3 ± 0.03 **	41
SEAQ	50	22.3 ± 0.02 **	25
	100	17.6 ± 0.03 **	40

SE, *S. edelbergii;* NH, n-hexane; AQ, aqueous; CH, chloroform; NB, n-BuOH; EA, EtOAc; CR, crude. Data were taken as mean ± SEM. Aspirin is the positive control; n = 3 with *p* ≤ 0.01 **.

## Data Availability

The data are available to the researchers upon request.

## Supplementary Materials

The following are available online, Figure S1: ^1^H-NMR of compound **UA,** Figure S2: ^13^C-NMR of compound **UA,** Figure S3: ESI-HRMS of **UA,** Figure S4: ^1^H-NMR of compound **OV,** Figure S5: ^13^C-NMR of compound **OV,** Figure S6: ESI-HRMS of **OV,** Figure S7: ^1^H-NMR of compound **OA,** Figure S8: ^13^C-NMR of compound **OA,** Figure S9: ESI-HRMS of **OA,** Figure S10: ^1^H-NMR of compound **BS,** Figure S11: ^13^C-NMR of compound **BS,** Figure S12: ESI-HRMS of **BS,** Figure S13: ^1^H-NMR of compound **MA,** Figure S14: ^13^C-NMR of compound **MA,** Figure S15: ESI-HRMS of **MA**, Figure S16: ^1^H-NMR of compound **TA,** Figure S17: ^13^C-NMR of compound **TA,** Figure S18: ESI-HRMS of **TA,** Figure S19: ^1^H-NMR of compound **FL-1,** Figure S20: ^13^C-NMR of compound **FL-1,** Figure S21: ESI-HRMS of **FL-1**, Figure S22: ^1^H-NMR of compound **FL-2,** Figure S23: ^13^C-NMR of compound **FL-2,** Figure S24: ESI-HRMS of **FL-2**, Figure S25: ^1^H-NMR of compound **FL-3,** Figure S26: ^13^C-NMR of compound **FL-3,** and Figure S27: ESI-HRMS of **FL-3.**

## Sample Availability

Samples of the compounds are not available from the authors.
