# Supplementary material for: Biomedical Applications of Scutellaria edelbergii Rech. f.: In Vitro and In Vivo Approach
Source: Molecules. 2021 Jun 19;26(12):3740. doi: 10.3390/molecules26123740 (PMC8234977; doi:10.3390/molecules26123740)
Supplement: Supplementary file 1 [file molecules-26-03740-s001.zip › molecules-1217187-supplementary.pdf]

Supplementary Material

Biomedical Applications of *Scutellaria edelbergii* Rech. f.: In Vitro and in Vivo Approach

Muddaser Shah [1,2], Waheed Murad [1,*], Najeeb Ur Rehman [2,*], Sobia Ahsan Halim [2], Manzoor Ahmed [3], Hazir Rehman [4], Muhammed Zahoor [5], Sidra Mubin [6], Ajmal Khan [2], Mohamed A. Nassan [7], Gaber El-Saber Batiha [8] and Ahmed Al-Harrasi [2,*]

[1] Department of Botany, Abdul Wali Khan University Mardan, Mardan 23200, Pakistan; muddasershah@awkum.edu.pk
[2] Natural and Medical Sciences Research Center, University of Nizwa, P.O. Box 33, Birkat Al Mauz 616, Nizwa, Oman; sobia_halim@unizwa.edu.om (S.A.H.); ajmalkhan@unizwa.edu.om (A.K.)
[3] Department of Chemistry, University of Malakand, Chakdara 18800, Pakistan; manzoorhej@yahoo.com
[4] Department of Microbiology, Abdul Wali Khan University Mardan, Mardan 23200, Pakistan; hazirrahman@awkum.edu.pk
[5] Department of Biochemistry, University of Malakand, Chakdara 18800, Pakistan; mohammadzahoorus@yahoo.com
[6] Department of Botany, Hazara University Mansehra, Mansehra 21310, Pakistan; shahhu123@gmail.com
[7] Department of Clinical Laboratory Sciences, Turabah University College, Taif University, P.O. Box 11099, Taif 21944, Saudi Arabia; m.nassan@tu.edu.sa
[8] Department of Pharmacology and Therapeutics, Faculty of Veterinary Medicine, Damanhour University, Damanhour 22511, Egypt; gaberbatiha@gmail.com
* Correspondence: waheedmurad@awkum.edu.pk (W.M.); najeeb@unizwa.edu.om (N.U.R.); aharrasi@unizwa.edu.om (A.A.-H.)

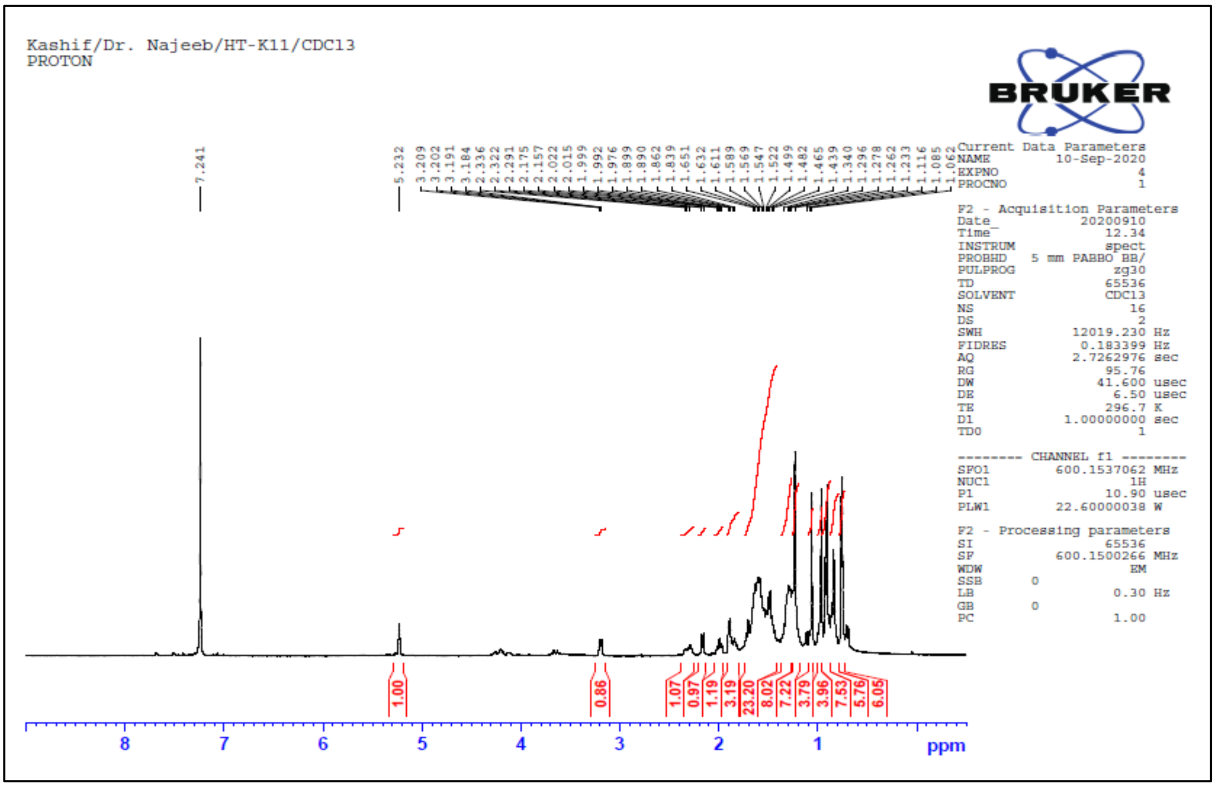

Figure S1: ^{1}H-NMR of compound **UA**

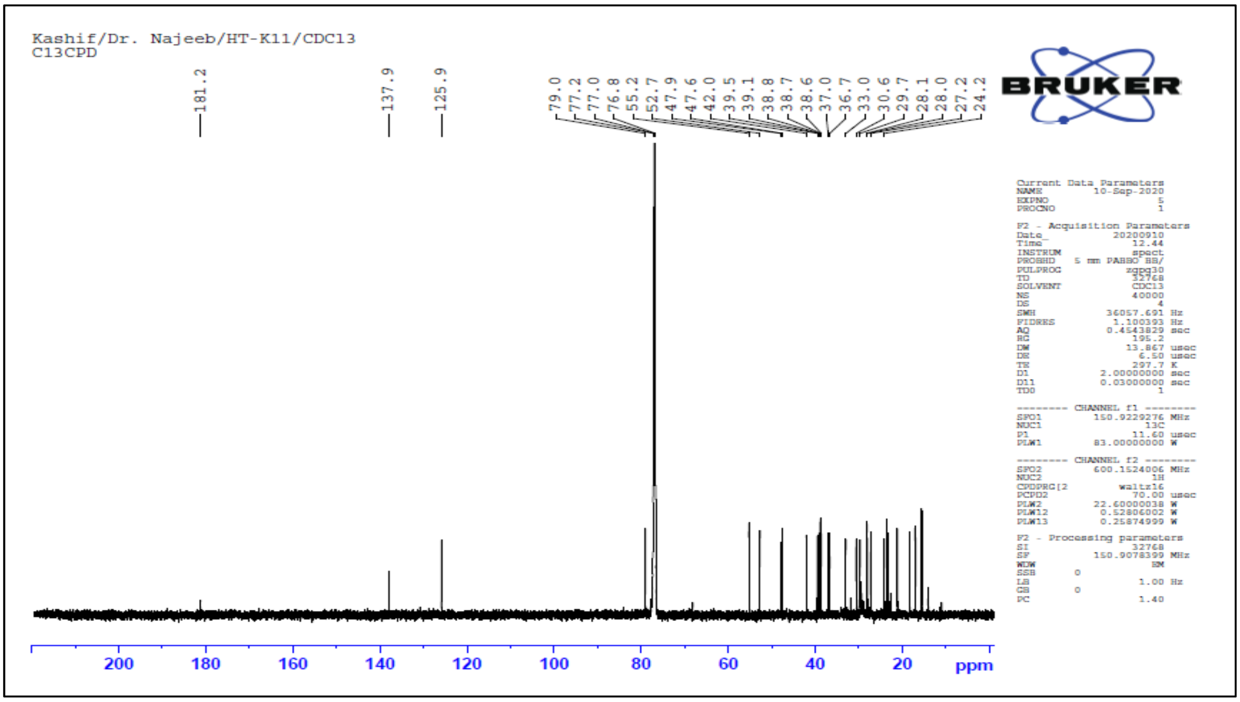

Figure S2: ^{13}C-NMR of compound **UA**

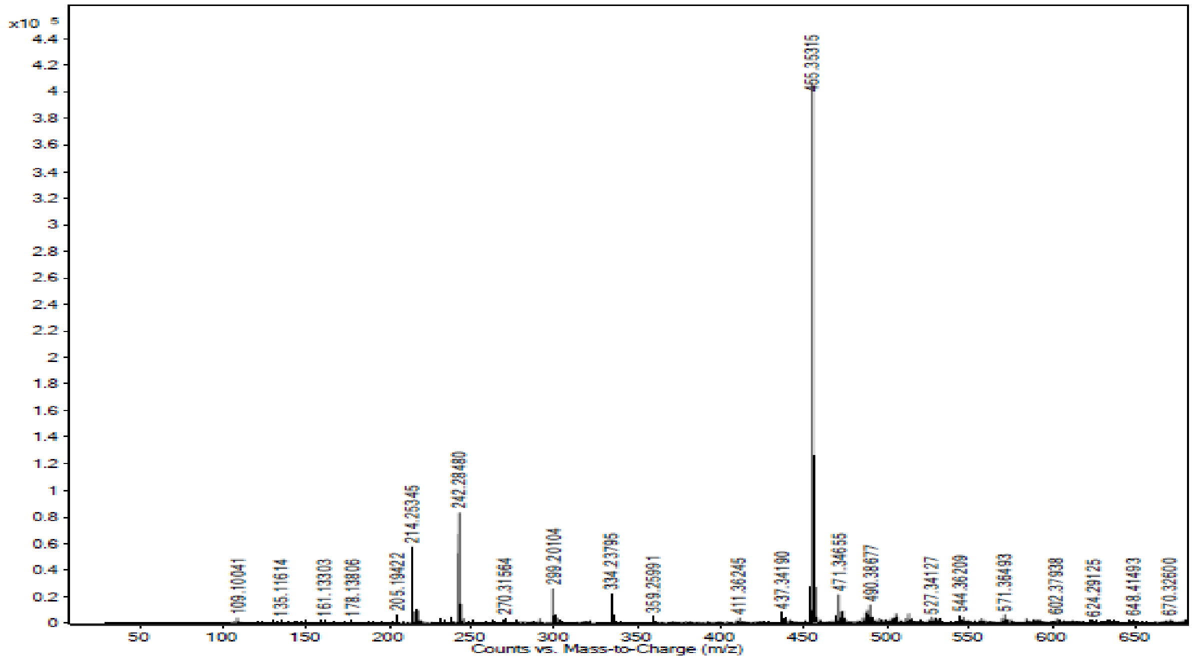

Figure S3: ESI-HRMS of **UA**

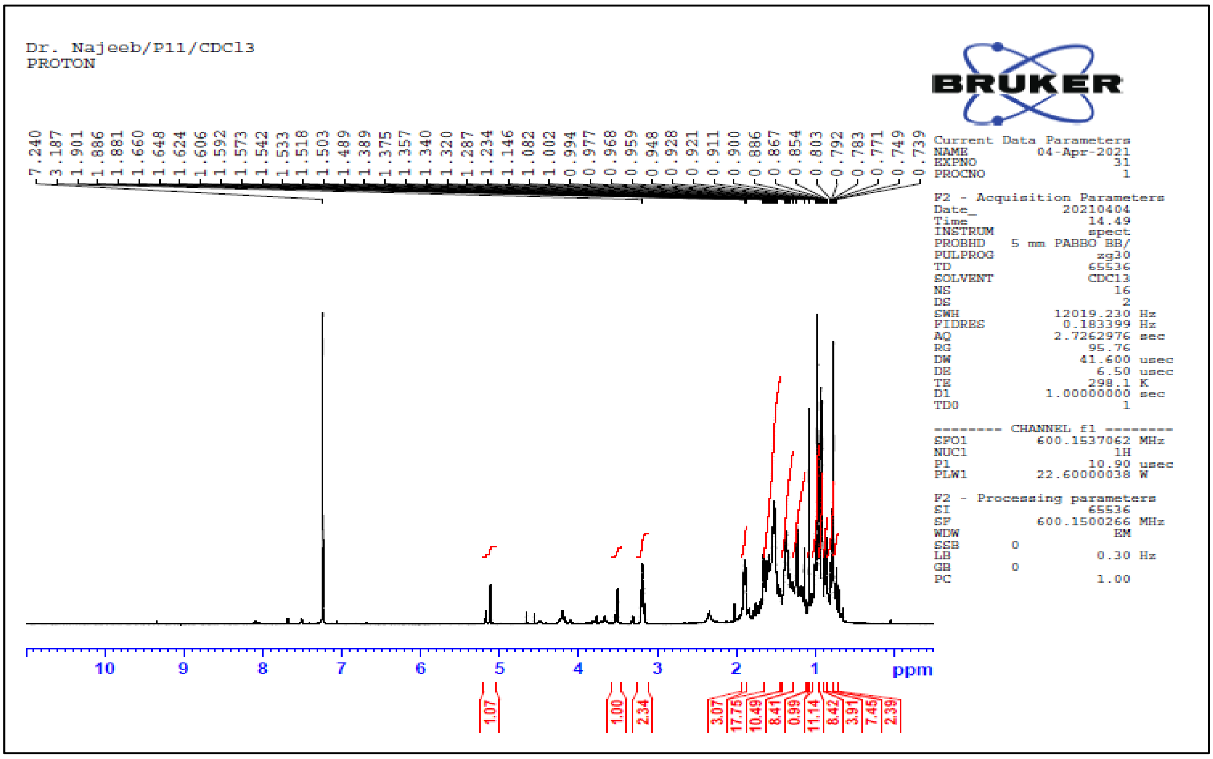

Figure S4: ^{1}H-NMR of compound **OV**

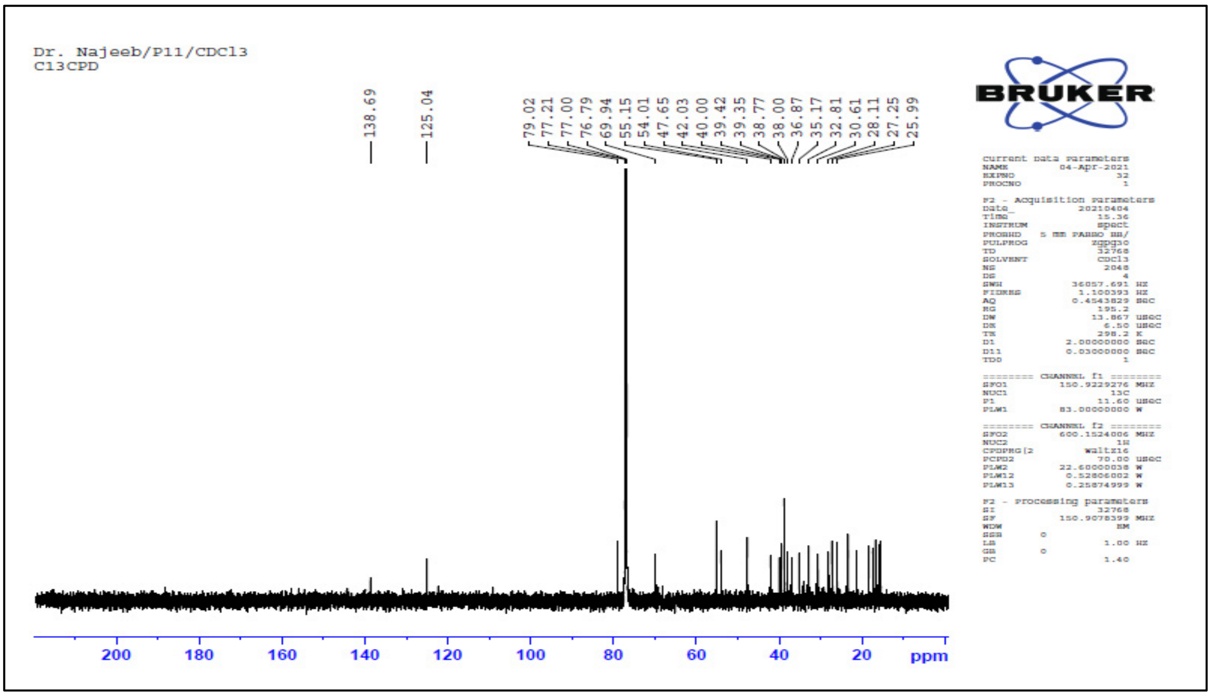

Figure S5: ^{13}C-NMR of compound **OV**

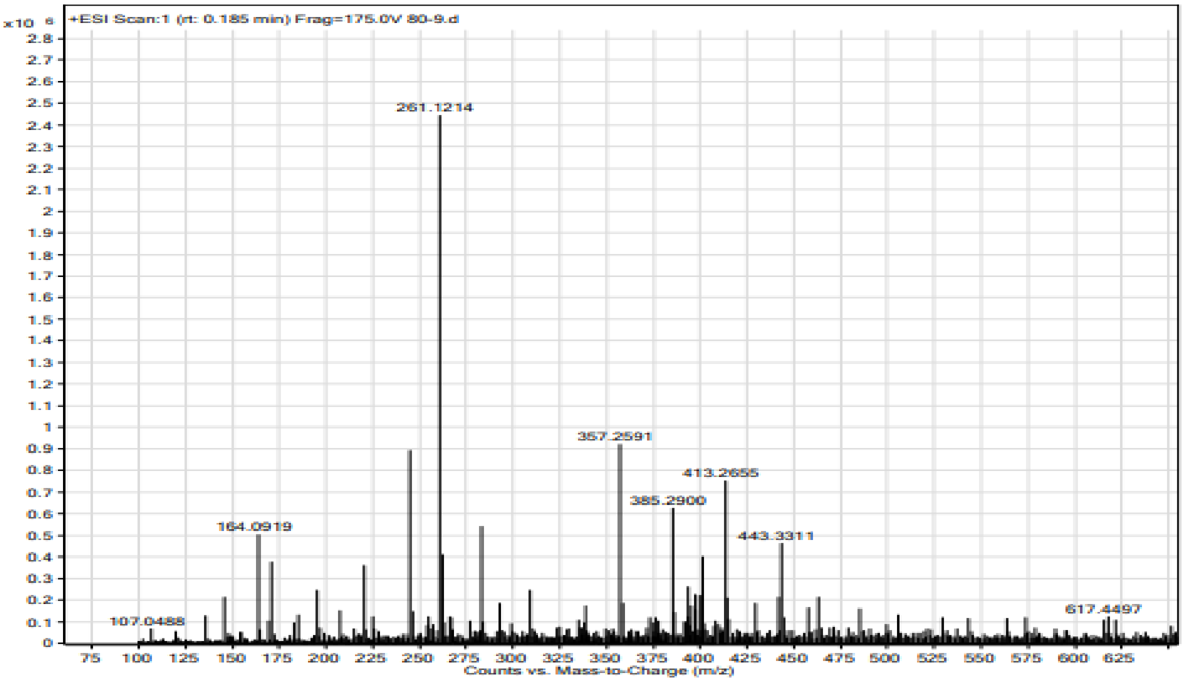

Figure S6: ESI-HRMS of **OV**

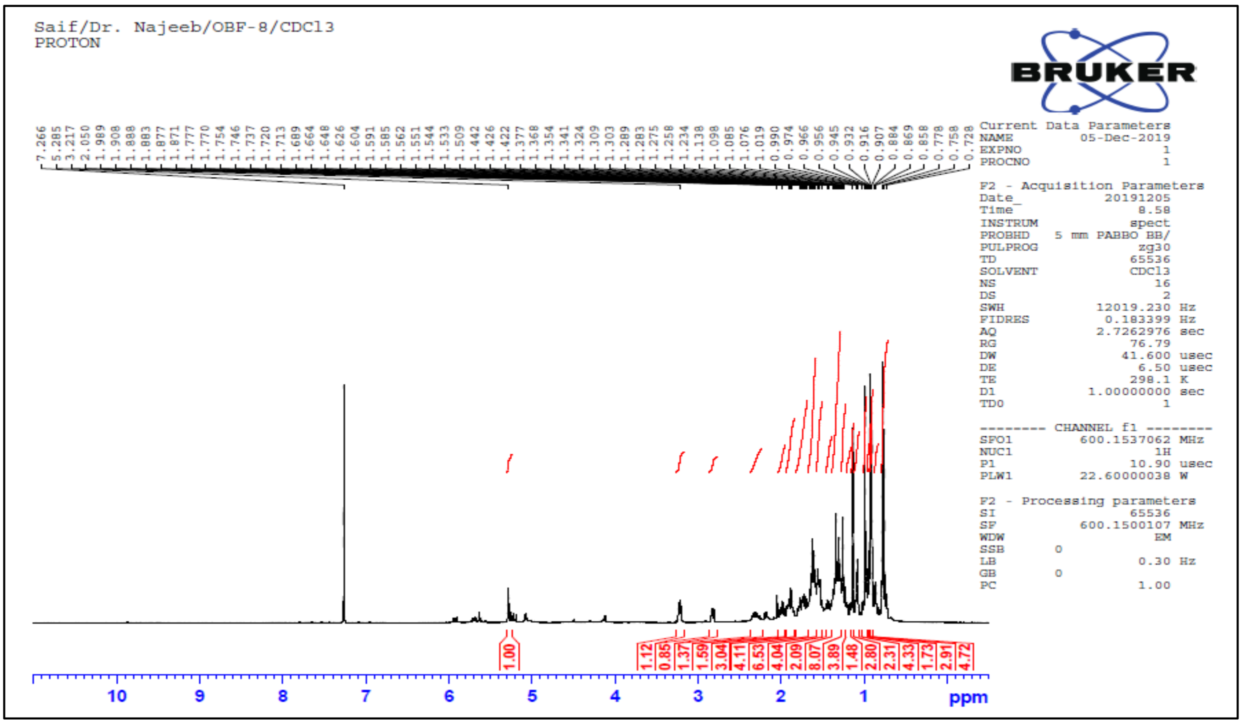

Figure S7: ^{1}H-NMR of compound **OA**

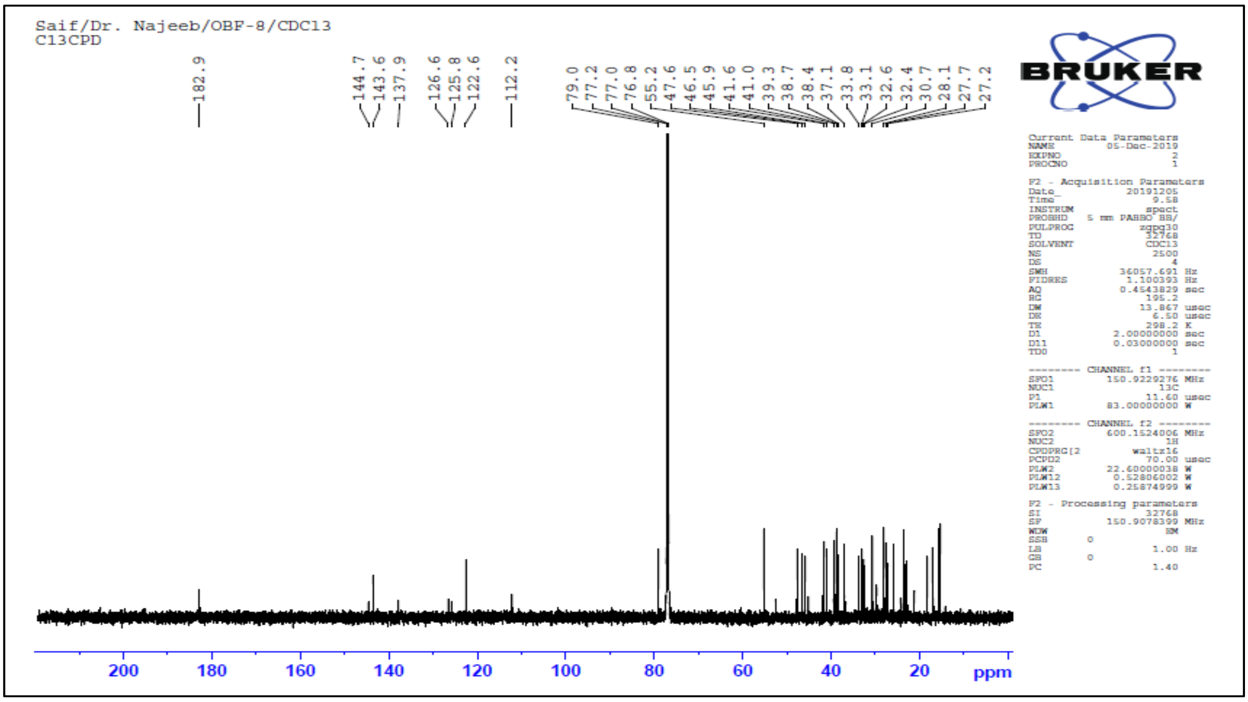

Figure S8: ^{13}C-NMR of compound **OA**

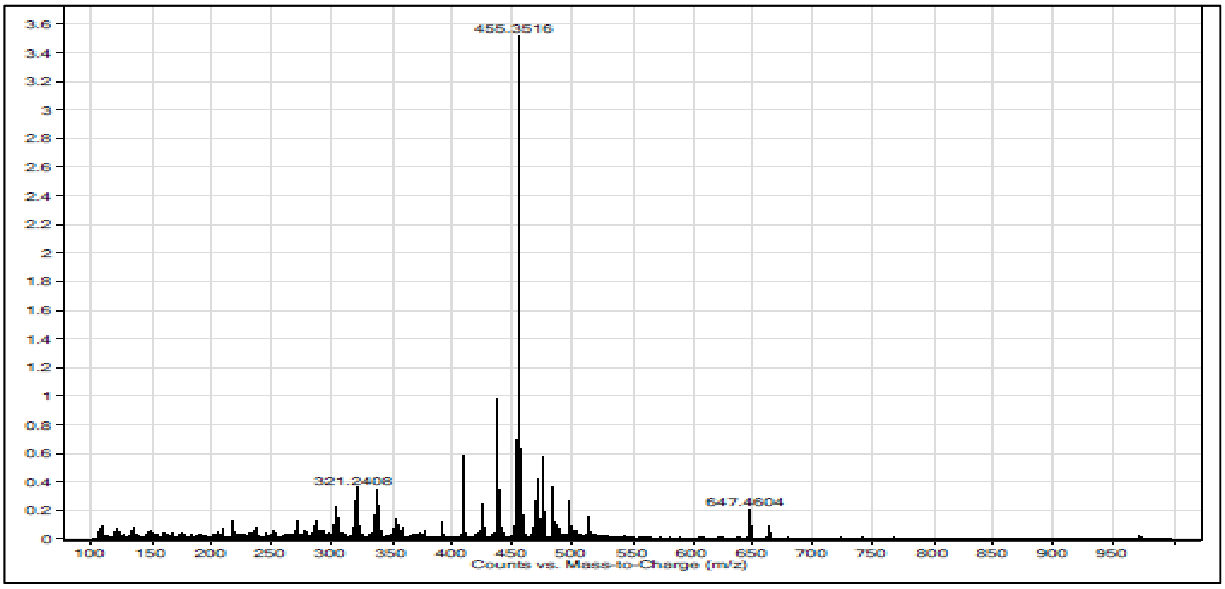

Figure S9: ESI-HRMS of **OA**

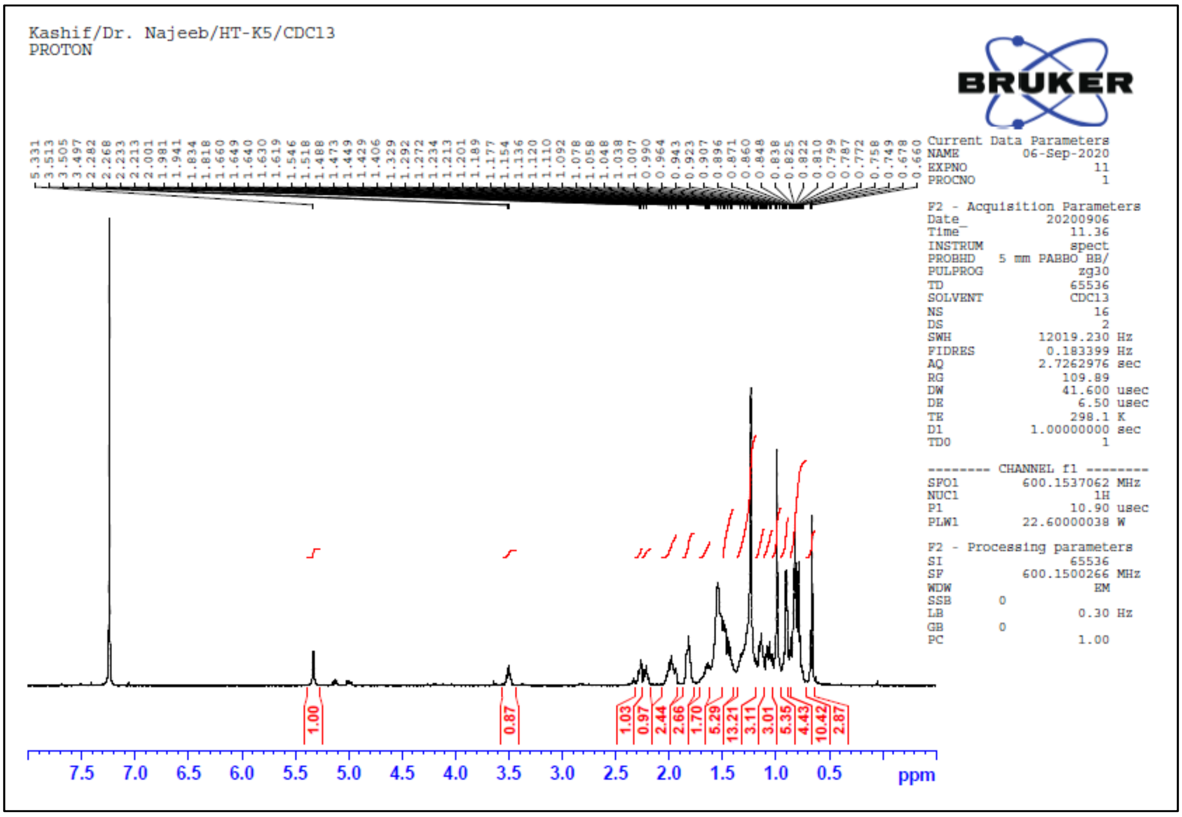

Figure S10: ^{1}H-NMR of compound **BS**

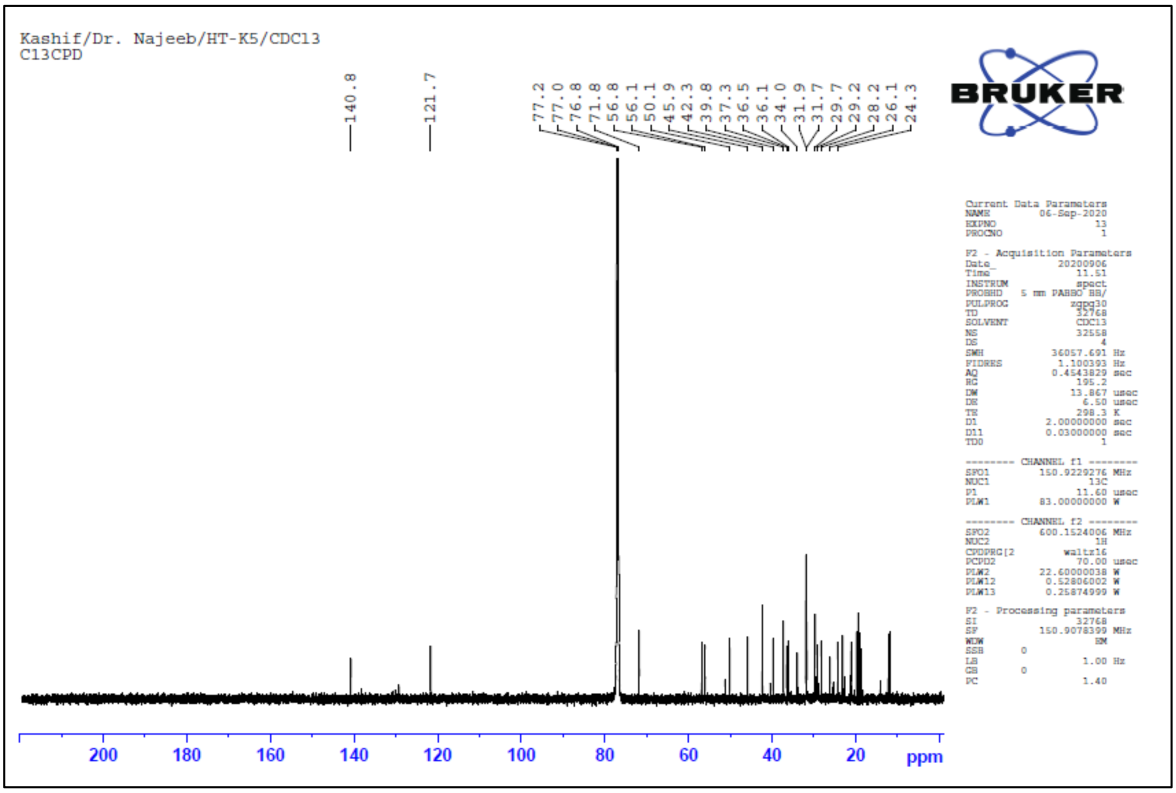

Figure S11: ^{13}C-NMR of compound **BS**

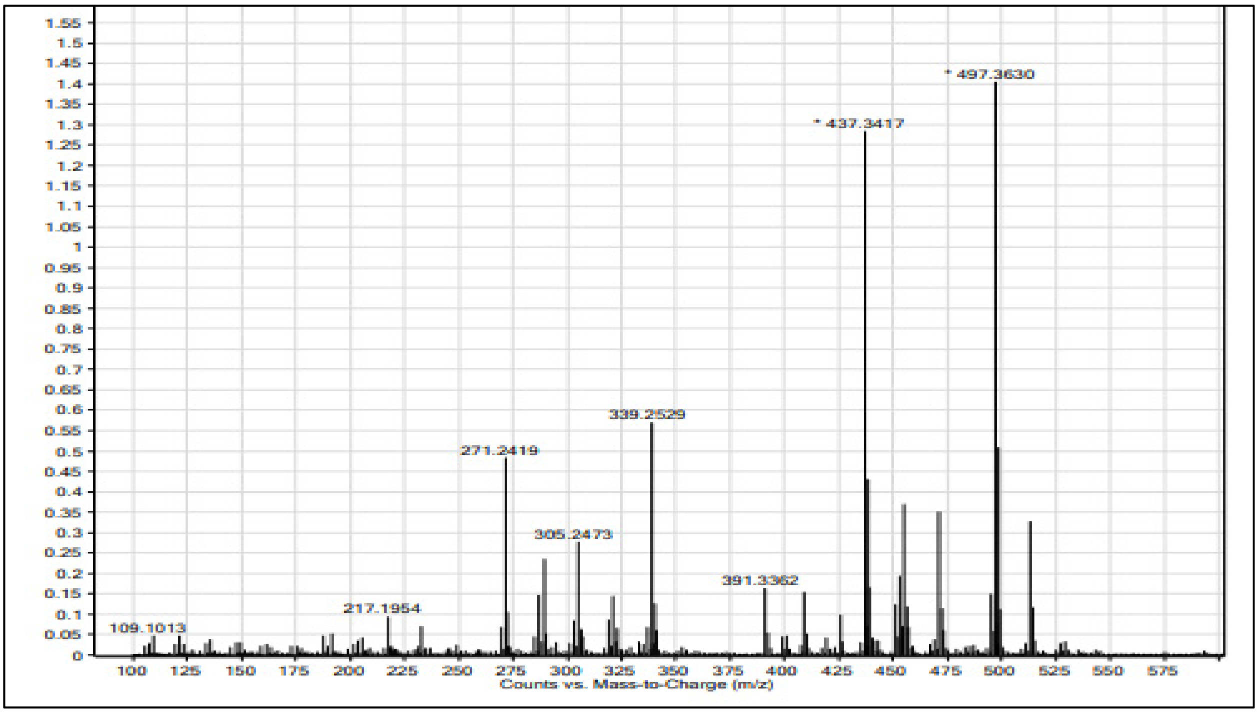

Figure S12: ESI-HRMS of **BS**

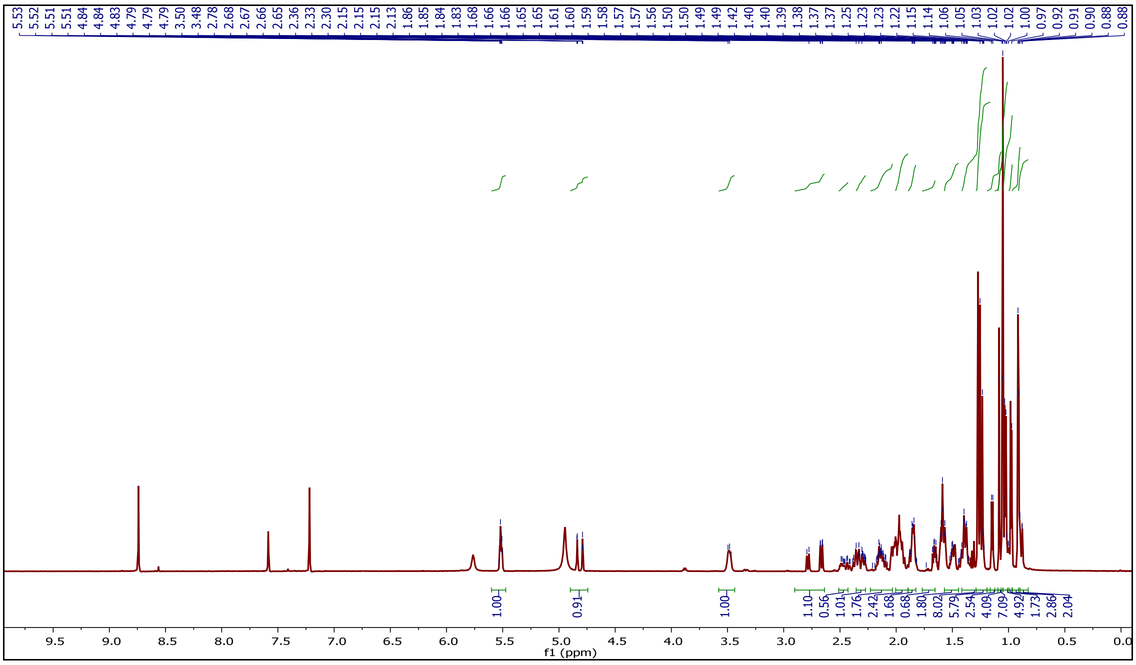

Figure S13: ^{1}H-NMR of compound **MA**

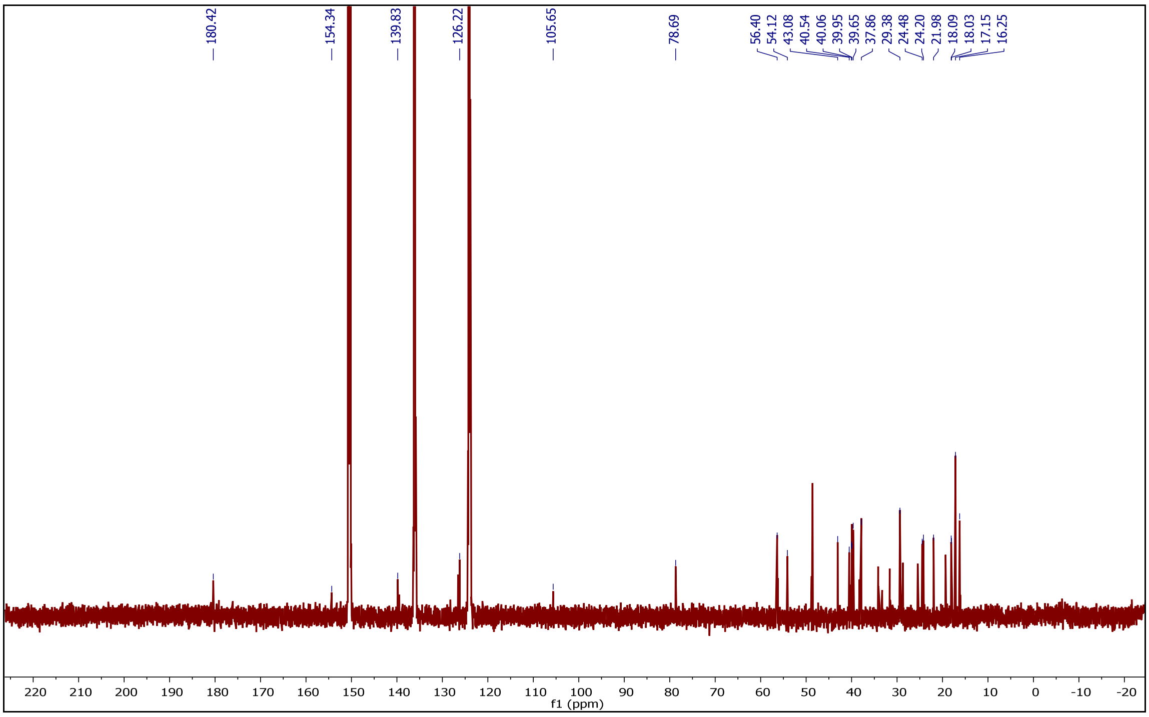

Figure S14: ^{13}C-NMR of compound **MA**

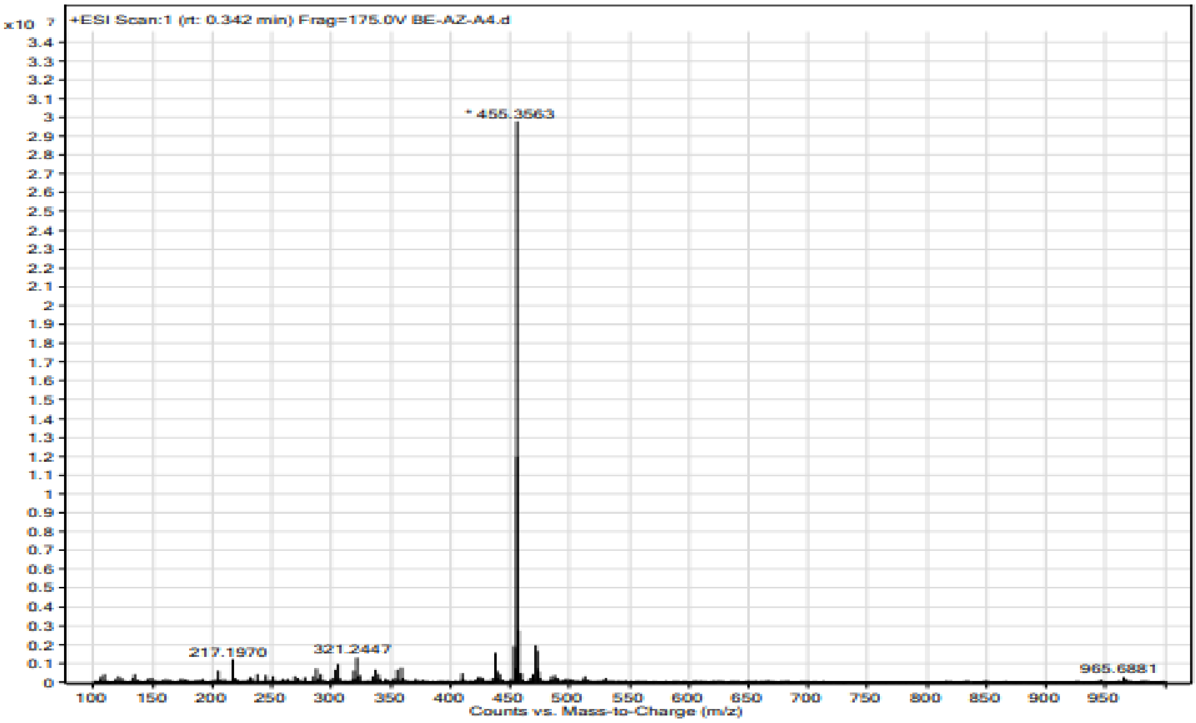

Figure S15: ESI-HRMS of **MA**

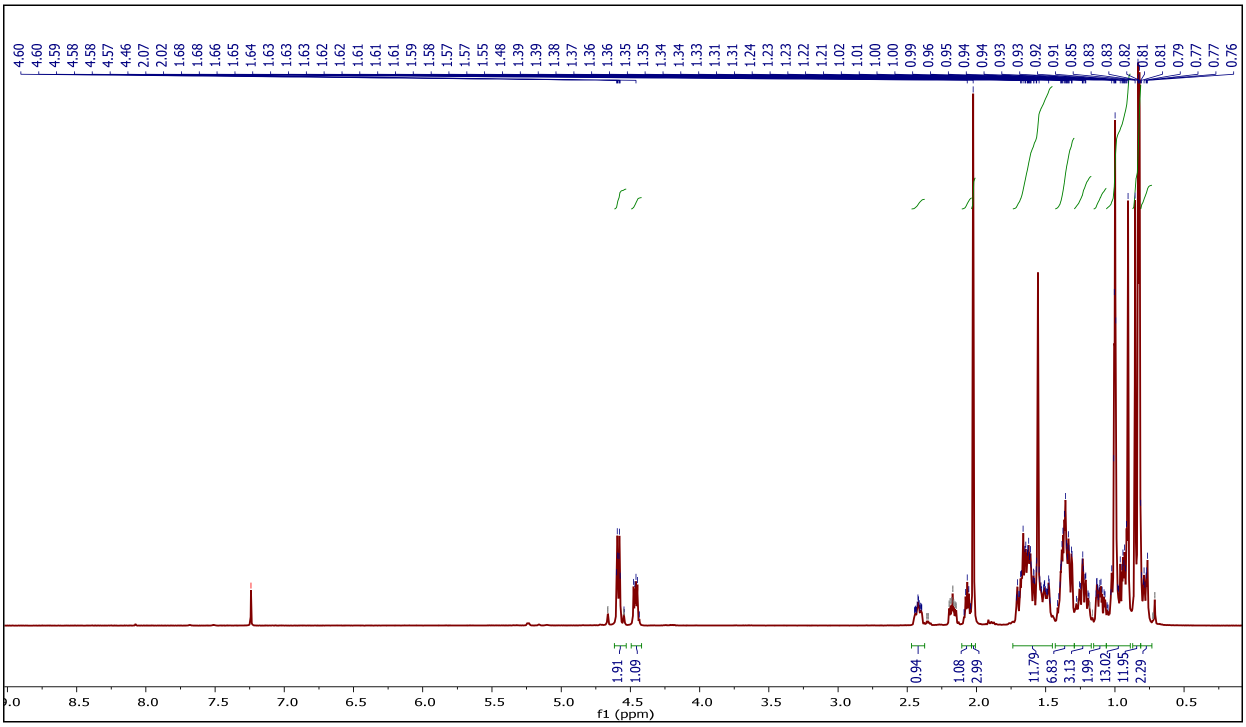

Figure S16: ^{1}H-NMR of compound **TA**

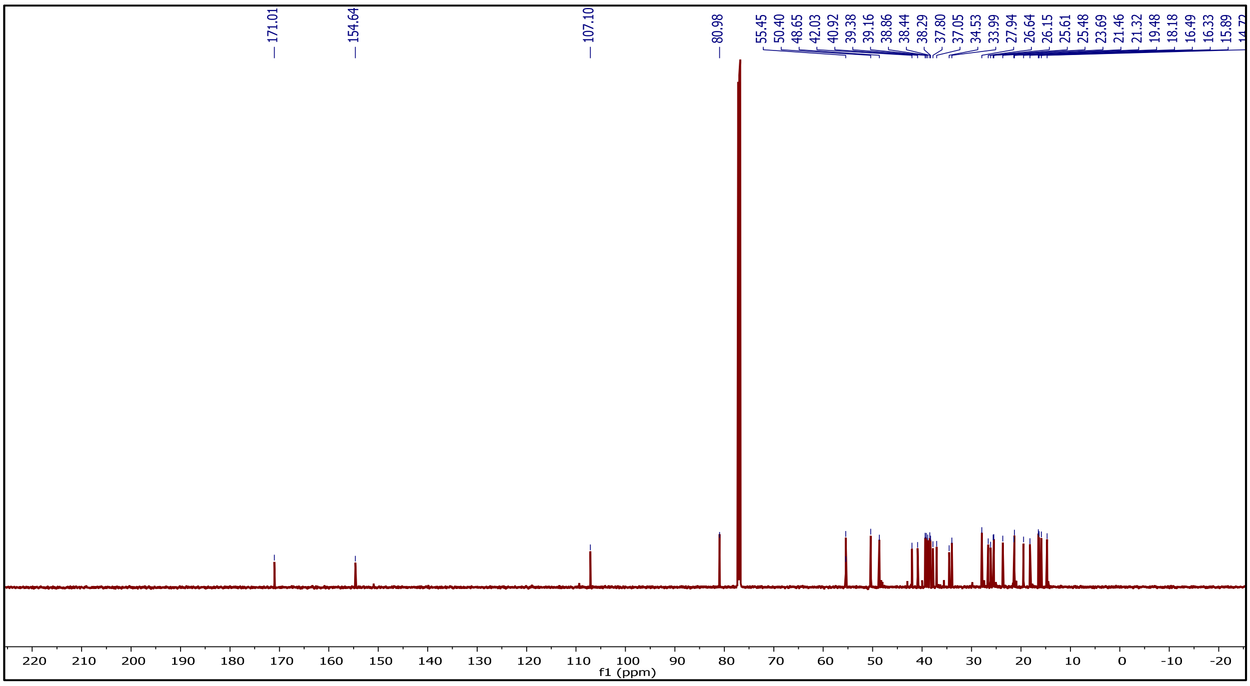

Figure S17: ^{13}C-NMR of compound **TA**

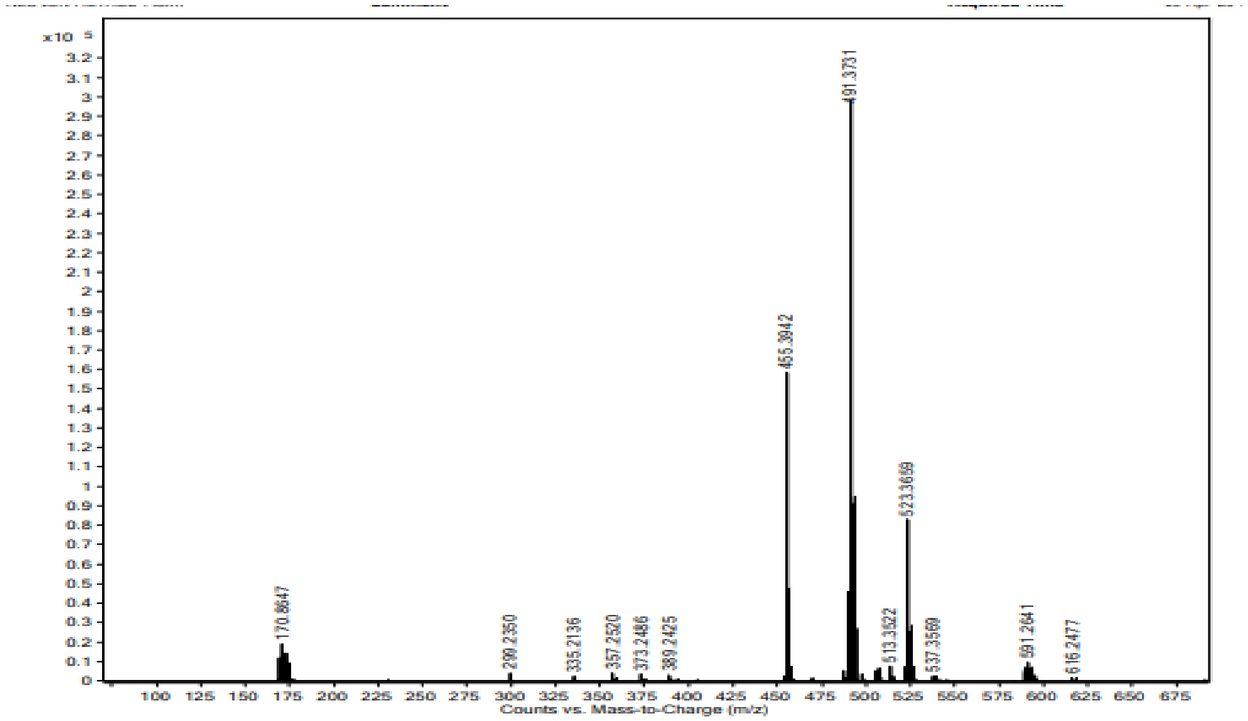

Figure S18: ESI-HRMS of **TA**

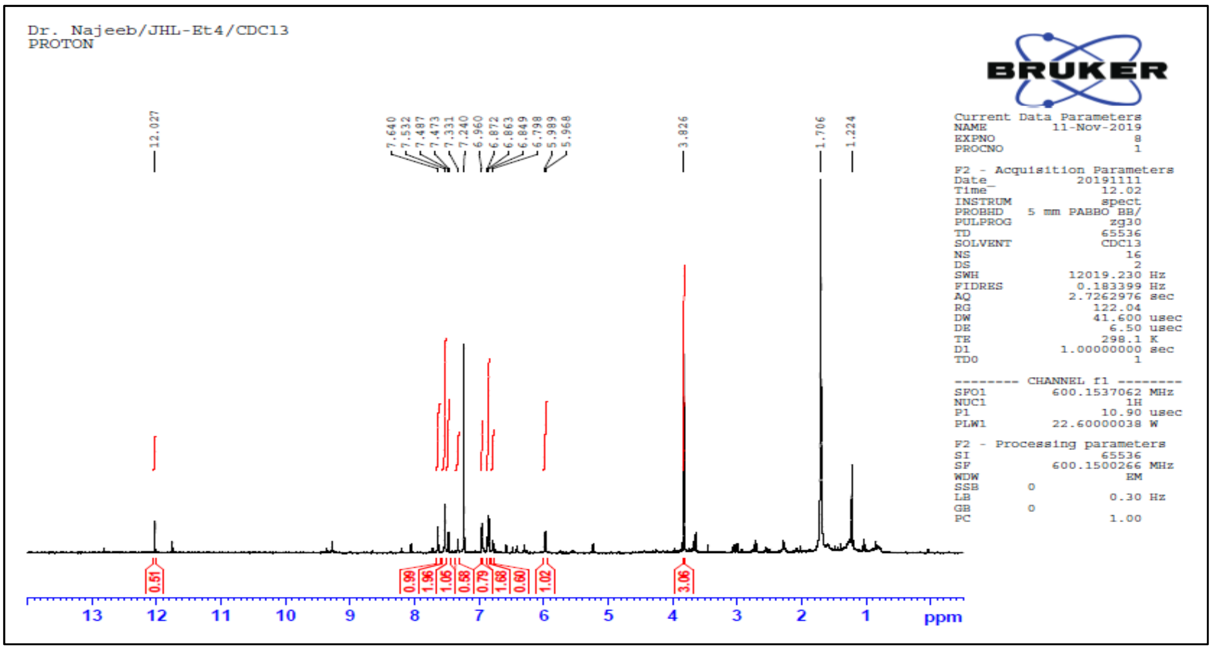

Figure S19: ^{1}H-NMR of compound **FL-1**

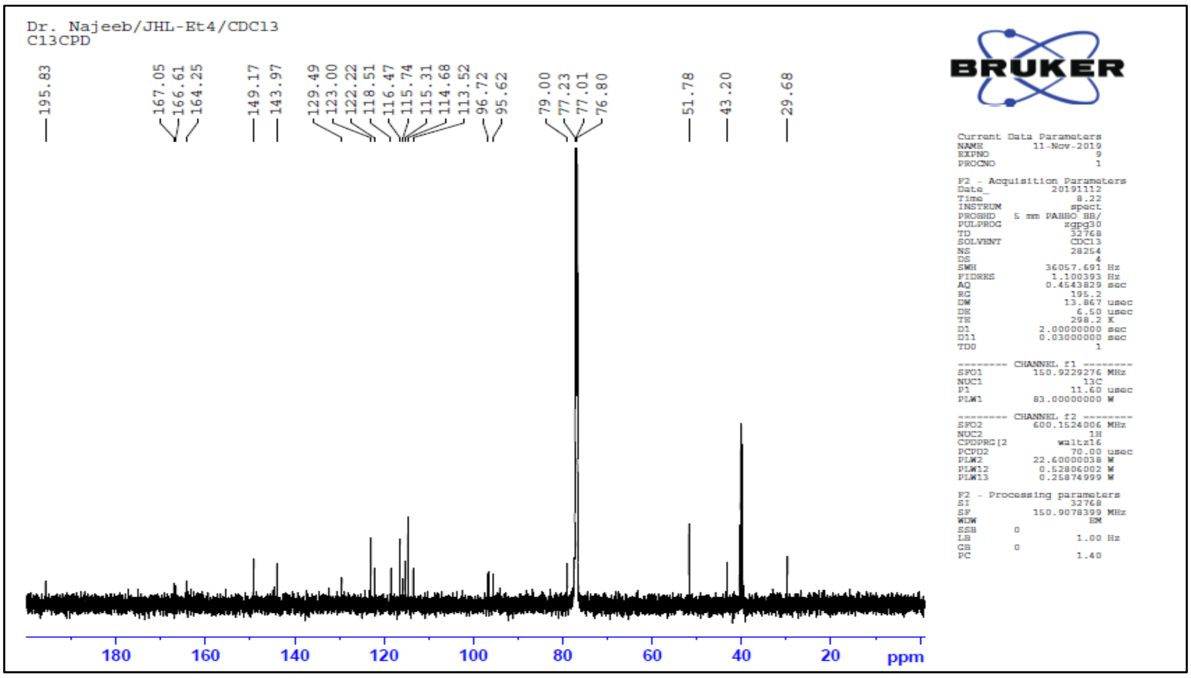

Figure S20: ^{13}C-NMR of compound **FL-1**

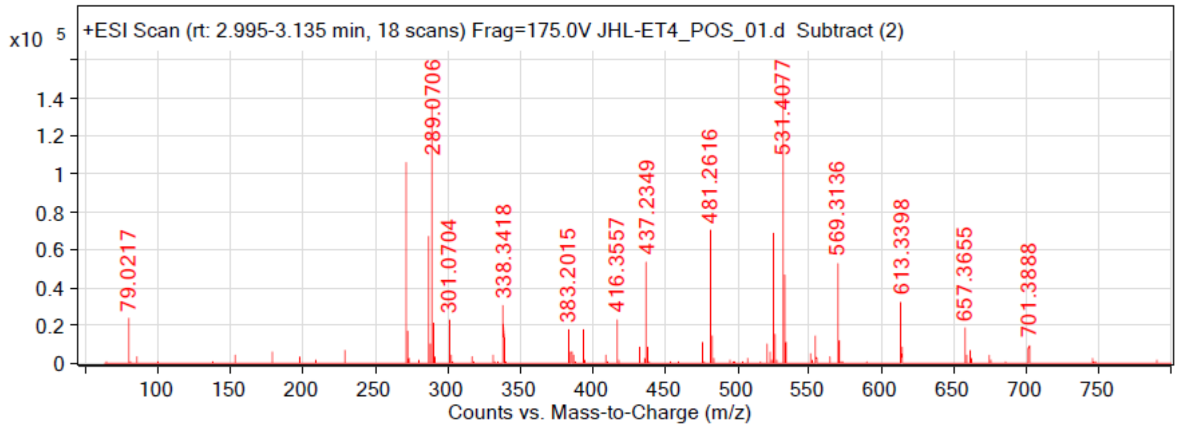

Figure S21: ESI-HRMS of **FL-1**.

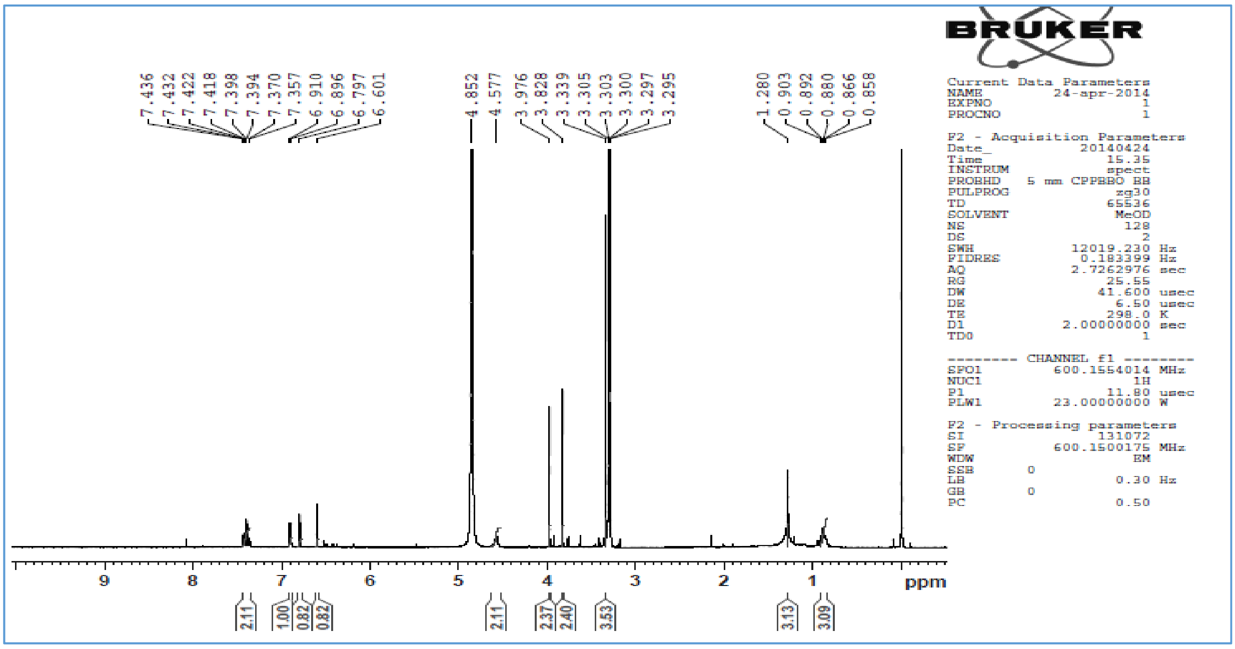

Figure S22: ^{1}H-NMR of compound **FL-2**

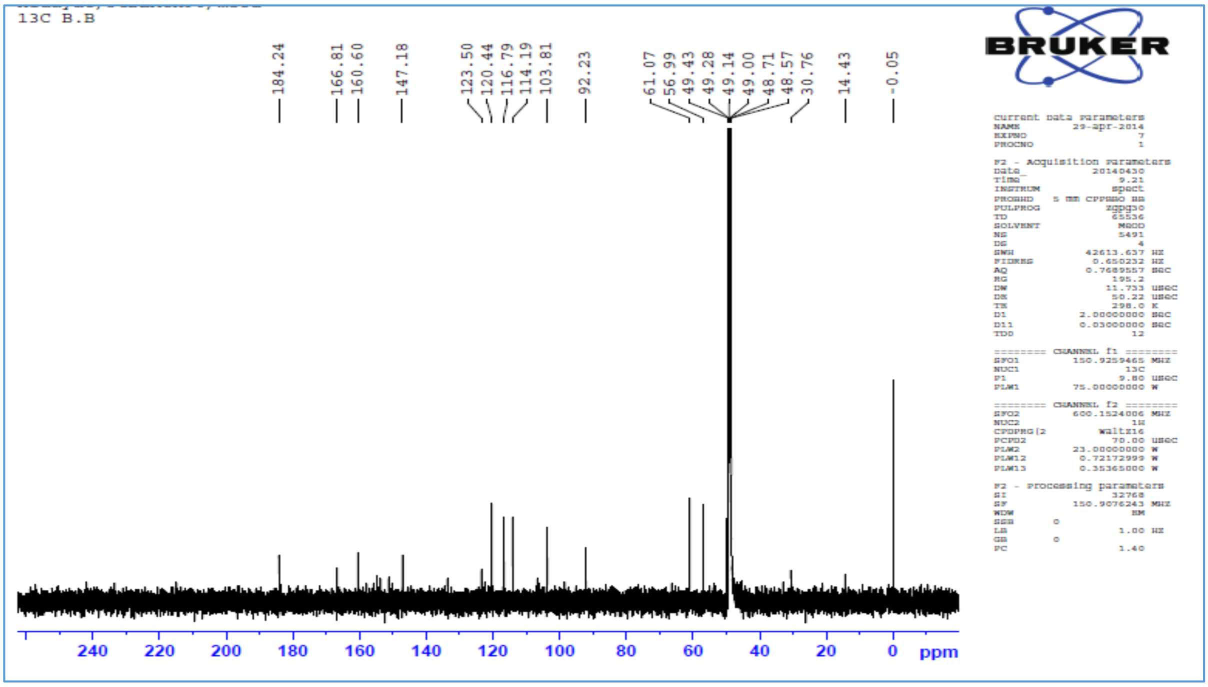

Figure S23: ^{13}C-NMR of compound **FL-2**

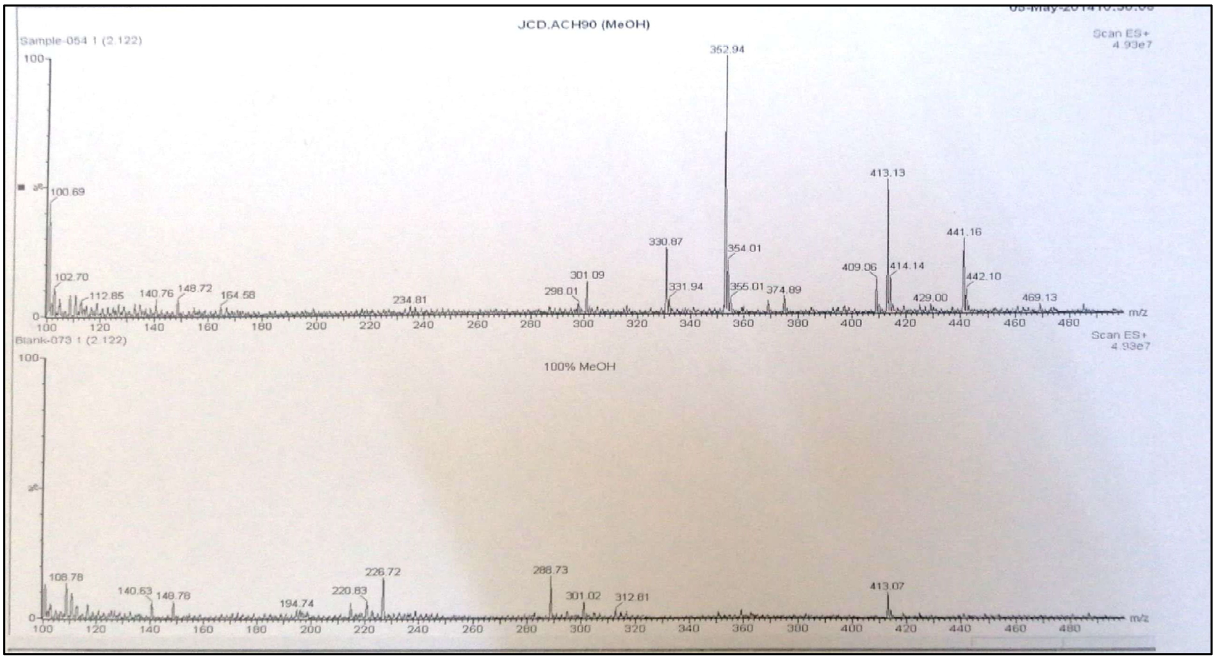

Figure S24: ESI-HRMS of **FL-2**

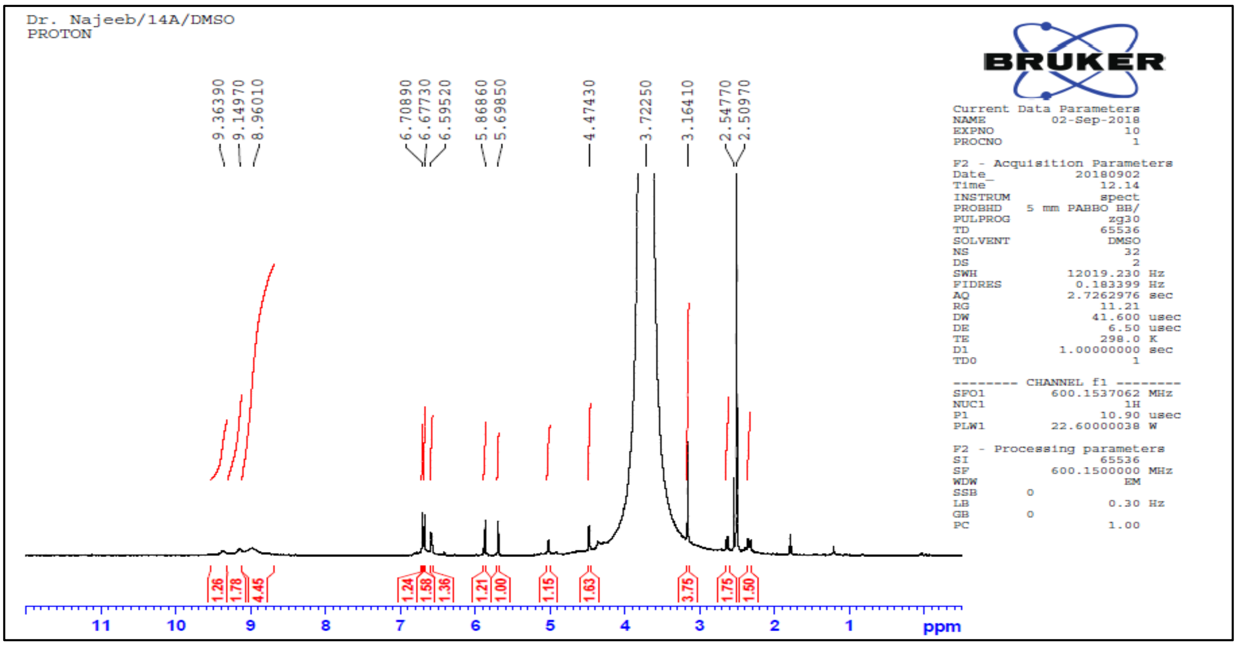

Figure S25: ^{1}H-NMR of compound **FL-3**

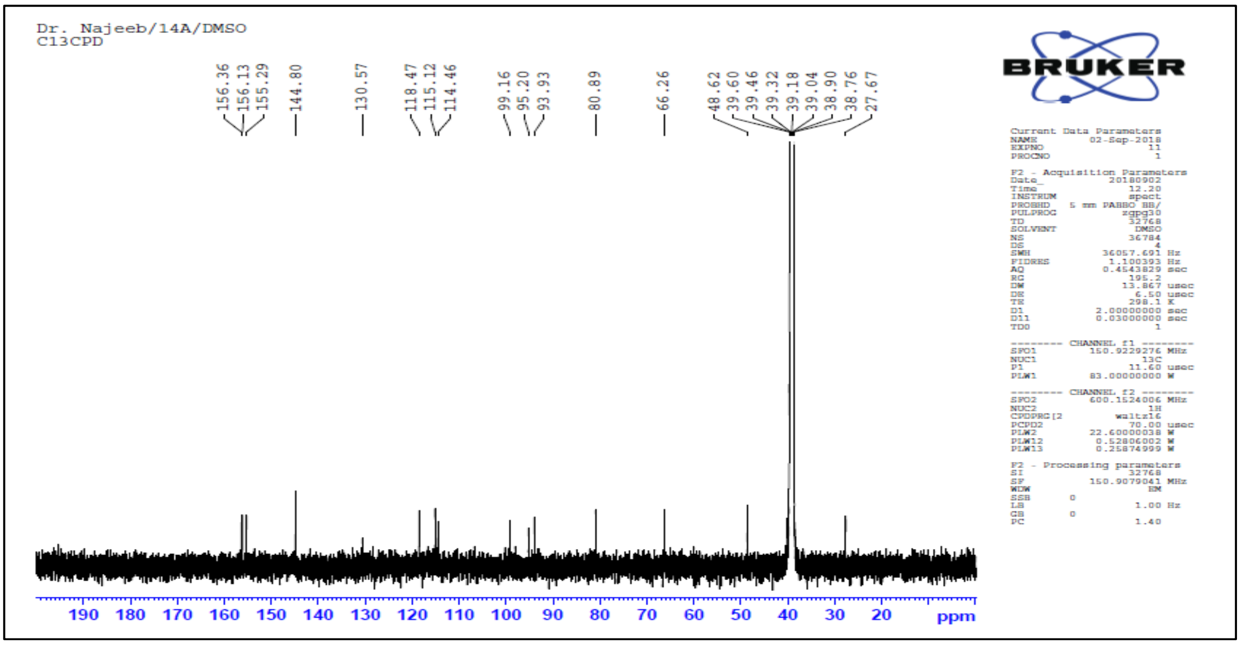

Figure S26: ^{13}C-NMR of compound **FL-3**

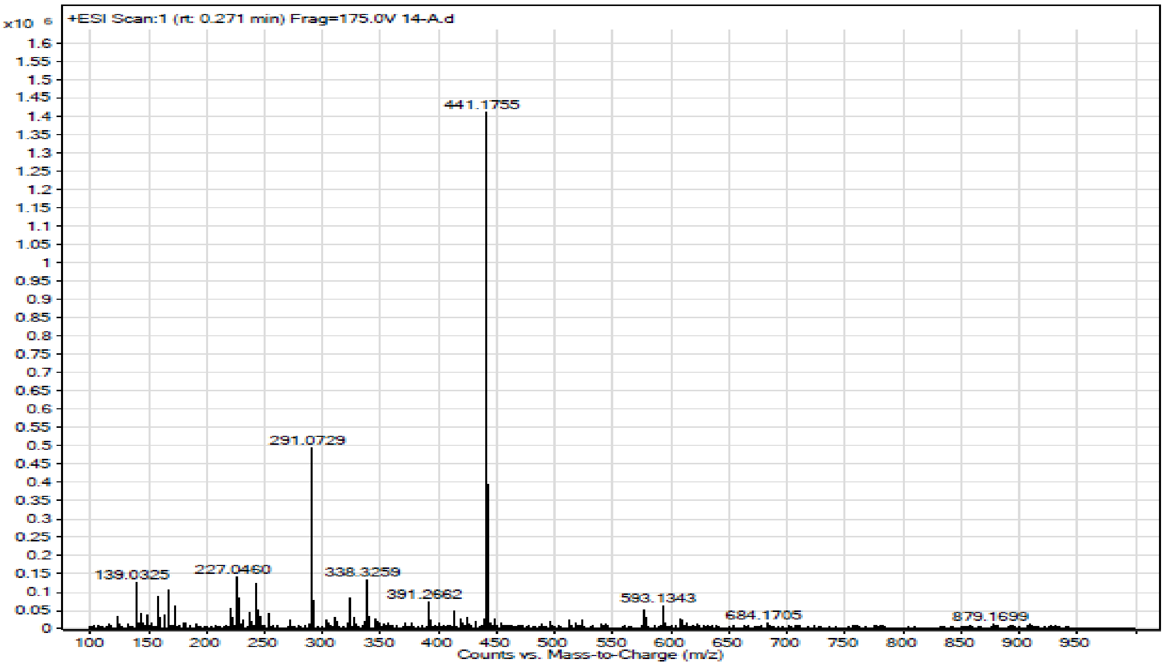

Figure S27: ESI-HRMS of **FL-3**
